# Effects of underlying morbidities on the occurrence of deaths in COVID-19 patients: A systematic review and meta-analysis

**DOI:** 10.7189/jogh.10.020503

**Published:** 2020-12

**Authors:** Md Mostaured Ali Khan, Md Nuruzzaman Khan, Md Golam Mustagir, Juwel Rana, Md Saiful Islam, Md Iqbal Kabir

**Affiliations:** 1 Department of Population Science and Human Resource Development, University of Rajshahi, Rajshahi, Bangladesh; 2 Department of Population Sciences, Jatiya Kabi Kazi Nazrul Islam University, Trishal, Mymensingh, Bangladesh; 3 Department of Public Health, North South University, Bashundhara, Dhaka, Bangladesh; 4 Planning, Monitoring and Research, Directorate General of Health Services (DGHS), Mohakhali, Dhaka, Bangladesh

## Abstract

**Background:**

Coronavirus disease 2019 (COVID-19), the most hectic pandemic of the era, is increasing exponentially and taking thousands of lives worldwide. This study aimed to assess the prevalence of pre-existing comorbidities among COVID-19 patients and their mortality risks with each category of pre-existing comorbidity.

**Methods:**

To conduct this systematic review and meta-analysis, Medline, Web of Science, Scopus, and CINAHL databases were searched using pre-specified search strategies. Further searches were conducted using the reference list of the selected studies, renowned preprint servers (eg, medRxiv, bioRxiv, SSRN), and relevant journals’ websites. Studies written in the English language included if those were conducted among COVID-19 patients with and without comorbidities and presented survivor vs non-survivor counts or hazard/odds of deaths or survivors with types of pre-existing comorbidities. Comorbidities reported in the selected studies were grouped into eight categories. The pooled likelihoods of deaths in each category were estimated using a fixed or random-effect model, based on the heterogeneity assessment. Publication bias was assessed by visual inspection of the funnel plot asymmetry and Egger’s regression test. Trim and Fill method was used if there any publication bias was found.

**Results:**

A total of 41 studies included in this study comprised of 27 670 samples. The most common pre-existing comorbidities in COVID-19 patients were hypertension (39.5%), cardiovascular disease (12.4%), and diabetes (25.2%). The higher likelihood of deaths was found among COVID-19 patients who had pre-existing cardiovascular diseases (odds ratio (OR) = 3.42, 95% confidence interval (CI) = 2.86-4.09), immune and metabolic disorders (OR = 2.46, 95% CI = 2.03-2.85), respiratory diseases (OR = 1.94, 95% CI = 1.72-2.19), cerebrovascular diseases (OR = 4.12, 95% CI = 3.04-5.58), any types of cancers (OR = 2.22, 95% CI = 1.63-3.03), renal (OR = 3.02, 95% CI = 2.60-3.51), and liver diseases (OR = 2.35, 95% CI = 1.50-3.69).

**Conclusions:**

This study provides evidence that COVID-19 patients with pre-existing comorbidities had a higher likelihood of death. These findings could potentially help health care providers to sort out the most susceptible COVID-19 patients by comorbidities, take precautionary measures during hospitalization, assess susceptibility to death, and prioritize their treatment, which could potentially reduce the number of fatalities in COVID-19.

The coronavirus disease 2019 (COVID-19) caused by the severe acute respiratory syndrome coronavirus 2 (SARS-CoV-2), a virus from the large coronavirus family, was first detected on December 31st, 2019 in Wuhan, China, and has now been deemed a global pandemic by the World Health Organization (WHO). The virus is highly transmissible (reproductive number: 1.6-6.5, doubling time: 6.4 to 7.4 days) [1], which is transmitted mainly through respiratory droplets (coughs or sneezes), close contact with the infected person [2,3], and touching surfaces or objects exposed to droplets [2,3]. As of July 27, 2020, 209 days since the virus was first detected, approximately 16.64 million people from 213 countries or territories have been infected with the virus [4,5]. Almost 0.66 million have already died [4], and about 1% of currently infected people are now in critical conditions [5]. To date, there is no specific medicine or vaccine for COVID-19; therefore, the majority of the affected countries are taking non-pharmaceutical interventions such as restriction in inhabitants' mobility, quarantine of suspected persons, isolation of infected persons, travel restrictions, and airport screening to reduce further infections [1,6,7].

The virus is equally transmissible in all ages; however, people who are now in critical conditions or who have died were more likely to be in the elderly population and had one or more morbidities [8-11]. Commonly reported comorbidities among patients who died from COVID-19 were hypertension, diabetes, cardiovascular disease, and cerebrovascular disease [8,12-14]. Notably, these comorbidities are independent causes of millions of annual deaths globally, 17.9 million deaths from cardiovascular diseases, 9 million deaths from cancers, 3.9 million deaths from respiratory diseases, and 1.6 million deaths from diabetes, according to a WHO report from 2018 [15]. People with one or more of these morbidities usually have a poor immune system, which increases their susceptibility to being infected, to reach critical condition, and even die of a secondary disease like COVID-19 [1,12,16-19]. Precautionary measures following COVID-19 in patients with one or more morbidities could be potential ways to combat its adverse outcomes and severities. Thus, we need to identify morbidities that are potentially increasing the risks of mortality, which are still lacking. Moreover, studies conducted among COVID-19 patients are highly varied with reported comorbidities and their associated likelihoods of mortality [8,10,20,21]. Therefore, a systematic review and meta-analysis is important for summarizing and enlisting of comorbidities, which the secondary COVID-19 infection could potentially lead to adverse outcomes including deaths as well as to know the likelihoods of deaths for each morbidity. However, the few reviews that have been conducted up until now, failed to serve these purposes and were limited to the studies’ settings and patients. For instance, detailed searches for this research explored four-reviews published, almost all were conducted based on evidences from China [14,22,23], which were limited to analyzing small scale hospital data [22], included only patients in severe condition [14,23] and failed to measure the effects of each comorbidity of death on COVID-19 [24]. No previous study has considered adequate global level evidence as well as the samples from all settings. To address these gaps, this study was conducted with two primary aims: (i) to summarize pre-existing comorbidities in patients with COVID-19, and (ii) to estimate the likelihood of mortality from COVID-19 with each category of pre-existing comorbidities. The study findings could help health care providers to take appropriate measures to control fatalities from this pandemic.

## METHODS

This systematic review and meta-analysis was conducted by following the Preferred Reporting for Systematic Review and Meta-Analysis (PRISMA) consensus statement [25]. Studies relevant to the COVID-19 disease among people who were admitted to the hospital with one or more pre-existing comorbidities were included.

### Search strategy

Four databases: Medline, Web of Science, Scopus, and CINAHL, were searched using pre-specified search strategies for each database and concluded on May 01, 2020. The search strategy consists of keywords on COVID-19 disease (COVID-19, 2019-nCoV, Coronavirus, SARS-CoV-2), pre-existing morbidity (comorbidity, morbidity), and patients’ survival status (mortality, death, died) combined using the Boolean operators (AND, OR). Details of the search strategies are presented in Table S1-S4 in the **Online Supplementary Document**. Additional searches were conducted using the reference list of the selected studies, relevant journals’ websites, and renowned preprint servers (medRxiv, bioRxiv, SSRN).

### Study selection criteria

All peer-reviewed and preprint (not-peer-reviewed) studies met the pre-specified inclusion criteria were included in this study.

### Inclusion criteria

Studies were included if they met the following inclusion criteria: (i) conducted for the hospitalized patients infected with COVID-19 with or without pre-existing comorbidities, (ii) presented survivor and non-survivor counts following COVID-19 among patients with or without pre-existing morbidity or presented hazard/risk/odds ratio of deaths or survival following COVID-19 with the types of morbidities, and (iii) published in the English language. Studies without complete information but met our inclusion criteria were included in the narrative review.

### Exclusion criteria

Studies were excluded if COVID-19 was reported among pregnant women or children (aged <18 years) because there are additional conditions or morbidities existing in these groups, such as pregnancy complications. We also excluded papers written in languages other than English. Additionally, we excluded review papers, correspondence, viewpoints, editorials, commentaries, and studies where no information related to previous morbidities were reported.

### Screening and review of articles

All articles identified through the specified search strategy that were compiled and screened in Endnote version X9 (Thomson Reuters, Philadelphia, PA, USA). Two investigators (MMAK, MGM) independently screened the included articles based on inclusion and exclusion criteria. Initially, the titles and abstracts of the included studies were screened for a full-text review. Finally, the full-text of each of the selected studies were reviewed by the same investigators. Any conflict of interest faced by the investigators was reported to the senior authors (MNK and MIK) and solve based on the group discussion.

### Data extraction and quality assessment

A data extraction form was designed, trialed, and modified to extract information from the selected studies. Two authors (MMAK and MGM) used the pre-designed form to extract information independently. The following information was extracted: study location, design, sample size, study population characteristics (eg, age, gender), and survivor vs non-survivor counts among COVID-19 patients with or without specific comorbidities. If available, the odds/risk/hazard ratio of deaths among COVID-19 patients with comorbidities were extracted against the types of morbidity. Disagreements reported in data extraction were reviewed and solved by the corresponding and senior authors (MNK and MIK). The Modified Newcastle-Ottawa scale, as part of the data extraction strategy, was used to assess the quality of selected studies [26]. The scale has been used commonly to assess the study quality included in the systematic review and meta-analysis [24,27,28].

### Statistical analysis

One or more pre-existing comorbidities among COVID-19 patients reported in the selected studies were grouped into eight broad categories based on the type of comorbidities. These were cardiovascular system diseases (hypertension, cardiovascular disease, arrhythmia, heart failure), immune and metabolic disorders (diabetes, immunosuppression, autoimmune disease, immunodeficiency, metabolic disorder), respiratory system diseases (chronic lung diseases, Chronic Obstructive Pulmonary Disease (COPD), acute respiratory distress syndrome, tuberculosis, etc.), cancer (malignancy, cancer, and tumor), cerebrovascular diseases (cerebrovascular disease, peripheral vascular disease), renal system diseases (chronic kidney disease, urinary disease), liver system diseases (chronic liver disease, cirrhosis, hyperlipidemia, Hepatitis B, etc.), and gastrointestinal system diseases (chronic digestive disorder, gastrointestinal disease). The case definition of these comorbidities is presented in **Table 1**. The odds ratios (ORs) of deaths with 95% confidence interval (95% CI) for the people exposed to a particular category of comorbidity as compared to COVID-19 patients unexposed to another comorbidity was estimated from the extracted raw data or reported ORs. We first used the Haldane correction (add constant 0.5 to each cell) for the studies in which the sample included in the exposed or unexposed group was zero (such as all exposed patients died or vice versa) [29-31]. We then used either a fixed effect or random effect model to estimate ORs, selected based on heterogeneity assessment. When the test of heterogeneity (*I^2^* statistics) was moderate (50%-74%) or high (≥75%), the pooled estimates of ORs were computed using the random-effects model [32]. Subgroup and meta-regression analyses were conducted for the groups where higher heterogeneity was reported. For this, pre-specified subgroups (types of morbidities, study country, study design, the mean age of the total sample, mean age of death sample) were used. Publication bias was assessed by visual inspection of the funnel plot asymmetry and Egger’s regression test [33]. When evidence of publication bias was found, the Trim and Fill method of sensitivity analysis was used to adjust potentially missing studies, and the effect size was recalculated accordingly [34]. Stata software version 15.1 (StataCorp. LP, College Station, TX, USA) was used for all analyses.

**Table 1:** Case definitions for underlying diseases sought in this systematic review and meta-analysis

Group	Disease	Case definition
**Cardiovascular system diseases**	Hypertension	Systolic blood pressure ≥140 mm Hg or diastolic blood pressure ≥90 mm Hg in two or more consecutive visits.
	Cardiovascular disease	A group of disorders of the heart and blood vessels
	Arrythmia	A disorder with the rate of the heartbeat (too slowl (≤60 beats/min), too fast (≥100 beats/min), or irregular rhythm)
	Heart failure	A condition when the heart muscle is unable pump enough blood to meet the body's blood and oxygen needs.
**Immune and metabolic disorders**	Diabetes	A disease that halts the body from producing the hormone insulin because of abnormal carbohydrate metabolism and increased blood glucose levels. Blood glucose level >200 mg/dL (11.1 mmol/L) after two hours of fasting indicates diabetes.
	Immunosuppression	Partial or complete suppression of the body's immune system to fight against the infections and other diseases.
	Autoimmune disease	A disorder in which the body’s immune system mistakenly attacks its own healthy tissues
	Immunodeficiency	The inability of the body to produce an immune response. In such condition the immune system's ability to fight infectious diseases and cancer is impaired or completely absent.
	Endocrine system disease	A hormonal imbalance in the body. It is determined through testing the blood and urine.
	Metabolic disorder	A disruption of normal metabolic processes because of one or more missing enzyme.
**Respiratory system diseases**	Chronic lung disease	A disorder that affects the lungs and other parts of the respiratory system
	Chronic obstructive pulmonary disease (COPD)	A disorder that restricts normal breathing and measured through Tiffeneau-Pinelli index (FEV1/FVC). The index value 50 or less refers moderate to severe stage of the COPD.
	Asthma	A disorder lead breathing difficulty. In such condition, the bronchial airways in the lungs become narrowed and swollen and Tiffeneau-Pinelli index reduced to 60 or less for moderate to severe stage of the Asthma.
	Acute respiratory distress syndrome (ARDS)	A respiratory failure characterized by rapid start of extensive inflammation in the lungs.
	Chronic bronchitis	The swelling and irritation of the bronchial tubes that can lead to chronic cough and breathing difficulties.
	Tuberculosis	A disorder caused by a bacterial infection (name, Mycobacterium tuberculosis) in the lungs.
	Pulmonary emphysema	A chronic condition in which the alveoli may be collapsed, damaged, narrowed, overinflated, or stretched.
**Any types of cancer**	Malignancy	An abnormal cell division without control that can invade nearby tissues.
	Cancer	A disorder in which any cells of the body begin to divide uncontrollably.
	Tumor	Uncontrolled growth occurs in solid tissue like muscle or bone and could cause cancer.
	Carcinoma	A specific type of cancer in epithelial tissue of the skin.
**Cerebrovascular system diseases**	Cerebrovascular disease	A disorder of the blood vessels and the arteries.
	Peripheral vascular disease	A disorder that makes the blood vessels narrow, block, or spasm outside of heart and brain. Ankle/brachial index (ABI) is used to measure this disorder where ABI>90 is normal and ABI<70 indicates moderate to severe condition.
**Renal system diseases**	Chronic kidney disease	Glomerular filtration rate (GFR)<60 or serum creatinine >1.1 mg/dL for women and >1.3 mg/dL for men refers to have chronic kidney disease.
	Urinary disease	Includes infection in urinary tract, kidney stone, bladder control problems, prostate problems.
**Liver system diseases**	Chronic liver disease	Alanine aminotransferase test (ALT)>40 U/L and aspartate aminotransferase test (AST)>40 U/L indicates chronic liver diseases
	Cirrhosis	A condition in which the liver fails to function properly due to long-term damage or liver disease like hepatitis, fatty liver
	Fatty liver disease	High fat builds up in liver.
	Hepatitis B	Virus liver infection caused by hepatitis B virus.
	Hyperlipidemia	High levels of fats (lipids) in the blood. Cholesterol >200 mg/dL, and triglycerides >150 mg/dL refers to hyperlipidemia
	Inflammatory disease	Erythrocyte sedimentation rate (ERS)>22 mm/h for man and >29 mm/h for women refers to the presence of inflammatory disease
**Gastrointestinal system diseases**	Chronic digestive disorder	Disorders that occur in the digestive tract. Some common disorders include irritable bowel syndrome, and lactose intolerance.
	Gastrointestinal disease	Diseases in gastrointestinal tract, namely the esophagus, large intestine, small intestine, and rectum

## RESULTS

**Figure 1:**
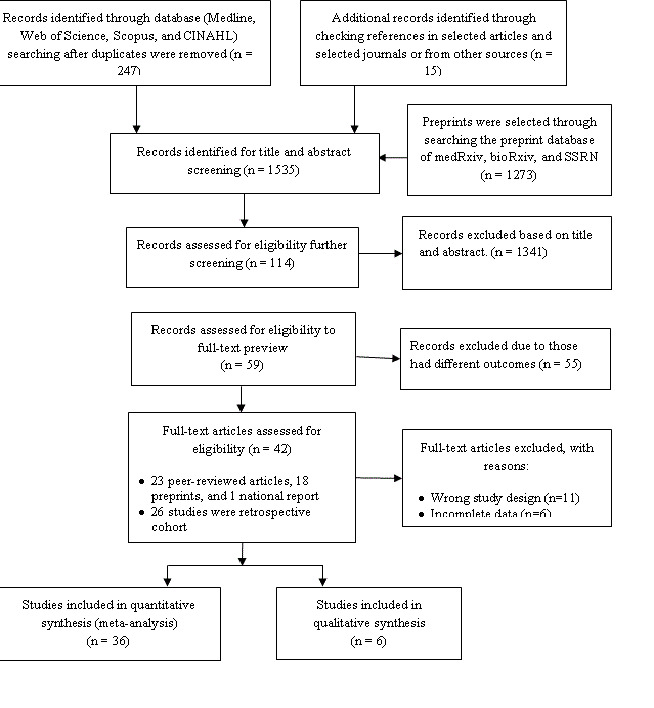
Schematic representation of studies included in the systematic review and meta-analysis using the PRISMA checklist and flow diagram.

A total of 247 articles were identified from the databases searched, and the additional 15 articles were identified by checking the reference list of the selected articles and the selected journals’ websites (**Figure 1**). Around 1273 articles were also initially identified from the preprint servers. Of the selected articles, 1341 articles were excluded after screening titles and abstracts, leaving 114 articles for full-text review for possible inclusion in this study. Of these, 55 articles were excluded based on the inclusion and exclusion criteria for the study sample (eg,, excluded pregnant or children), and 11 articles were excluded for study types (eg, review papers, correspondence, viewpoints, editorials, commentaries), and six articles were excluded for entirely incomplete data. One article was excluded because it has been retracted by the published journal. A total of 41 articles were finally selected for this study; 35 articles were included in the meta-analysis, and the remaining six articles were synthesized narratively.

### Study characteristics

A summary of the 41 selected articles is represented in **Table 2**. A total of 22 of the selected 41 articles were published in peer-reviewed journals, and 18 articles were published in pre-print servers. One of the selected studies was a national report for Australia. The majority of these studies were retrospective in nature (32), along with eight prospective studies. The selected studies comprised of 27 677 COVID-19 patients, 7558 (42.5%) of them had pre-existing one or more comorbidities, 38.4% of patients had undergone critical care, and 4795 (17.3%) of them died. Their average age was 60.9 ± 7.9 years, and 60.3% of them were male. The mean age at death was 70.4 ± 4.9 for the patients who died of COVID-19. A total of 35 selected studies presented death counts following COVID-19 among patients with or without one or more specific comorbidities. Four included studies (Du et al. [66], Zhang et al. [67], Kim et al. [68], and Yao et al. [69]) were conducted only for dead COVID-19 patients and reported the status of pre-existing comorbidities before their deaths. All studies were of moderate to high quality (Table S6-S7 in the **Online Supplementary Document**).

**Table 2:** Background characteristics of the selected studies and hospitalized sample (n = 27 760)

Sl	Authors	Article type	Study country	Publication date	Study type	Total sample (n)	Total death sample, n (%)	Mean age (±SD, or 95% CI)	Mean age at death (±SD, or 95% CI)	Male sample (%)	Female sample (%)	Prevalence of any forms of pre-existing morbidities among COVID-19 disease patients*, n (%)	ICU admission following COVID-19 disease ^†^ (%)
**Studies included in the meta-analysis**													
1.	Guan et al. [8]	Peer reviewed	China	26 Mar 2020	Retrospective	1590	50 (3.1)	48.9 ± 16.3	-	57.3	42.7	399 (25.1)	6.2
2.	Cao et al. [35]	Peer reviewed	China	2 Apr 2020	Prospective	102	17 (16.7)	54 (37-67)	72 (63-81)	52.0	48.0	47 (46.1)	17.6
3.	Chen et al. [36]	Peer reviewed	China	31 Mar 2020	Retrospective	274	113 (41.2)	62 (44-70)	68 (62-77)	62.0	38.0	133 (48.5)	-
4.	Deng et al. [37]	Peer reviewed	China	20 Mar 2020	Retrospective	225	109 (48.4)	54	69 (62-74)	55.1	44.9	127 (56.4)	-
5.	Yang et al. [10]	Peer reviewed	China	24 Feb 2020	Retrospective	52	32 (61.5)	59.7 ± 13.3	64.6 ± 11.2	67.0	33.0	21 (40.4)	38.5
6.	Wu et al. [38]	Peer reviewed	China	13 Mar 2020	Retrospective	201	44 (21.9)	51 (43-60)	-	63.7	36.3	-	26.4
7.	Chen et al. [20]‡	Peer reviewed	China	11 Apr 2020	Retrospective	55	19 (34.5)	74	77	61.8	38.2	37 (67.3)	-
8.	Zhou et al. [21]	Peer reviewed	China	9 Mar 2020	Retrospective	191	54 (28.3)	56 (46-67)	69 (63-76)	62.0	38.0	91 (47.6)	26.0
9.	Yuan et al. [39]	Peer reviewed	China	19 Mar 2020	Retrospective	27	10 (37.0)	60 (47-69)	68 (63-73)	45	55	13 (48.1)	-
10.	Chen et al. [40]	Preprint	China	30 Mar 2020	Retrospective	123	5 (4.1)	51 (35-66)	-	40.7	59.3	15 (12.2)	-
11.	Caramelo et al. [41]§	Preprint	China	25 Feb 2020	Retrospective	-	-	-	-	51.4	48.6	-	-
12.	Ren et al. [42]¶	Peer reviewed	China	11 Feb 2020	Retrospective	5	1 (20.0)	53.6	61	60.0	40.0	2 (40.0)	100
13.	Australian Govt. [43]	Report	Australia	5 Apr 2020	Retrospective	810	69 (8.5)	59.5	79 (74-84)	-	-	-	13
14.	Shi et al. [44]	Peer reviewed	China	25 Mar 2020	Retrospective	416	57 (13.7)	64 (21-95)	-	49.3	50.7	-	-
15.	Zhang et al. [11]	Peer reviewed	China	15 Apr 2020	Retrospective	663	25 (3.8)	56 (44-69)	67.1 (61-78)	48.4	51.6	-	14.2
16.	Du et al. [45]	Peer reviewed	China	8 Apr 2020	Prospective	179	21 (11.7)	57.6 ± 13.7	70.2 ± 7.7	54.2	45.8	-	-
17.	Wang et al [46]‡	Peer reviewed	China	15 Mar 2020	Retrospective	339	65 (19.2)	71 ± 8	76 (70-83)	49.0	51.0	-	23.6
18.	Fu et al. [47]	Preprint	China	16 Mar 2020	Retrospective	200	34 (17.0)	-	-	49.5	50.5	161 (80.5)	25.5
19.	Paranjpe et al. [48]	Preprint	USA	26 Apr 2020	Prospective	1078	310 (28.8)	65 (54-76)	75 (64-85)	70.5	29.5	-	43.3
20.	Cummings et al. [49]	Preprint	USA	20 Apr 2020	Prospective	257	86 (33.5)	62 (51-72)	-	66.1	33.9	-	100.0
21.	Guo et al. [50]‡	Preprint	China	14 Apr 2020	Retrospective	118	51 (43.2)	71.6	73.1 ± 7.3	44.9	55.1	-	-
22.	Zhu et al. [51]	Preprint	China	8 Apr 2020	Retrospective	325	17 (5.2)	45 (34-61)	63 (57-76)	42.2	57.8	69 (21.2)	18.5
23.	Yin et al. [52]¶	Preprint	China	7 Apr 2020	Retrospective	112	52 (46.4)	66 (56-76)	70 (62-78)	68.7	31.3	71 (63.4)	100.0
24.	Sun et al. [53]¶	Preprint	China	6 Apr 2020	Retrospective	69	57 (82.6)	66 (59-73)	66 (62-77.5)	67.0	33.0	40 (58)	100.0
25.	Luo et al. [54]	Preprint	China	24 Mar 2020	Retrospective	403	100 (24.8)	56 (39-68)	71 (65-80)	47.9	52.1	175 (43.4)	50.9
26.	Zhang et al. [55]	Preprint	China	23 Mar 2020	Retrospective	315	47 (14.9)	57 (44-66)	66 (61-72)	55.6	44.4	103 (32.7)	56.5
27.	Solis et al. [56]	Preprint	Mexico	25 Apr 2020	Cross-sectional	7497	650	46	-	57.9	42.1	3726 (49.7)	-
28.	Yao et al. [57]	Peer reviewed	China	24 Apr 2020	Retrospective	108	12 (11.1)	52 (37-58)	65 (51-73.5)	39.8	60.2	25 (23.1)	15.7
29.	Zangrillo et al. [58]	Peer reviewed	Italy	23 Apr 2020	Prospective	73	17 (23.3)	61 (54-69)	-	83.6	16.4	-	45.2
30.	Yan et al. [59]	Peer reviewed	China	06 Apr 2020	Retrospective	193	108 (56.0)	64 (49-73)	70 (62-78)	59.1	40.9	94 (48.7)	47.7
31.	Tedeschi et al. [60]	Peer reviewed	Italy	27 Apr 2020	Prospective	609	179 (29.4)	68 (55-80)	-	68.0	32.0	-	-
32.	Nikpouraghdam et al. [61]	Peer reviewed	Iran	21 Apr 2020	Retrospective	2878	239 (8.3)	55.5 ± 15.2	65.4 ± 13.7	66.0	34.0	323 (10.9)	
33.	Benelli et al. [62]	Preprint	Italy	30 Apr 2020	Retrospective	411	72 (17.5)	66.8 ± 16.4	81.1 ± 7.5	66.6	33.4	256 (62.3)	6.8
34.	Levy et al. [63]	Preprint	USA	30 Apr 2020	Retrospective	4933	1185 (24.0)	-	-	58.4	41.6	-	-
35.	Sneep et al. [64]	Preprint	UK	29 Apr 2020	Retrospective	200	28 (14.0)	63.4 ± 17.8	76 ± 14	57.5	42.5	-	11.0
**Studies included in the narrative review**													
36.	Grasselli et al. [65]†¶	Peer reviewed	Italy	6 Apr 2020	Retrospective	1591	405 (25.6)	63 (56-70)	-	82.0	18.0	1043 (65.6)	100.0
37.	Du et al. [66]	Peer reviewed	China	7 Apr 2020	Prospective	109	109 (100)	70.7 ± 10.9	70.7 ± 10.9	67.9	32.1	85 (77.9)	46.8
38.	Zhang et al. [67]	Preprint	China	27 Feb 2020	Retrospective	82	82 (100)	72 (65-80)	72.5 (65-80)	65.9	34.1	62 (75.6)	17.1
39.	Kim et al. [68]	Preprint	Korea	20 Apr 2020	Retrospective	101	101 (100)	76 ± 10.3	76 ± 10 · 3	52.5	47.5	100 (99.1)	84.2
40.	Yao et al. [69]¶	Preprint	China	13 Mar 2020	Retrospective	55	55 (100)	70.7 ± 13.5	70.7 ± 13.5	67.0	33.0	43 (78)	100.0
41.	Cheng et al. [70]	Peer reviewed	China	20 Mar 2020	Prospective	701	113 (16.1)	63 (50-71)	-	52.4	47.6	297 (42.4)	10.4
	**Total or average**					**27 670**	**4800 (17.3)**	**60.9 ± 7.9**	**70.4 ± 4.9**	**60.3**	**39.7**	**7558 (42.5)**	**38.4**

SD – standard deviation, CI – confidence interval

*Complete data available for 17794 patients, and missing sample had incomplete information or directly indicated the likelihood of mortality or did not report people count with any forms of existing comorbidities. Sample with missing value were excluded from percentage calculation.

†Included only the patients in ICU or in critical condition with or without pre-existing morbidities, and data was available for 10154 patients.

‡Included only aged or elderly people as sample.

§Not included in total calculation as the study was a secondary data analysis.

¶Included only the patients in ICU or in critical condition.

Sample included only death patients; study of Tedeschi et al [60] had all patients more than one comorbidities.

### Prevalence of pre-existing morbidity among COVID-19 patients

Distribution of the type of morbidity presented in **Table 3**. Approximately 39.5% of the total COVID-19 patients reported that they had hypertension, 25.2% had diabetes, 12.4% had cardiovascular disease, 5.8% had chronic lung disease, 2.6% had COPD, and 4.4% had chronic kidney disease.

**Table 3:** Percentage distribution of comorbidities among patients reported in admission COVID-19 infection*

Pre-existing morbidities	Distribution of comorbidities for total patients	(%)**¶**
**Cardiovascular system diseases**	**9112**	**53.2**
Hypertension	6758	39.5
Cardiovascular disease†	2128	12.4
Arrhythmia	89	0.5
Heart failure	137	0.8
**Immune and metabolic disorders**	**4581**	**26.8**
Diabetes	4318	25.2
Immunosuppression	179	1.0
Endocrine system disease	69	0.4
Autoimmune disease	8	0.0
Immunodeficiency	3	0.0
Metabolic disorder	4	0.0
**Respiratory system diseases**	**1849**	**10.8**
Chronic lung disease	991	5.8
Chronic obstructive pulmonary disease (COPD)	449	2.6
Asthma	376	2.2
Acute respiratory distress syndrome (ARDS)	11	0.1
Chronic bronchitis	10	0.1
Tuberculosis	9	0.1
Pulmonary emphysema	3	0.0
**Any types of cancer**	**248**	**1.4**
Malignancy	135	0.8
Cancer	92	0.5
Tumor	17	0.1
Carcinoma	4	0.0
**Cerebrovascular system diseases**	**205**	**1.2**
Cerebrovascular disease‡	196	1.1
Peripheral vascular disease	9	0.1
**Renal system diseases**	**781**	**4.6**
Chronic kidney disease	760	4.4
Urinary disease	21	0.1
**Liver system diseases**	**172**	**1.0**
Chronic liver disease	52	0.3
Cirrhosis	33	0.2
Fatty liver disease	15	0.1
Hepatitis B	49	0.3
Hyperlipidemia	17	0.1
Inflammatory disease	6	0.0
**Gastrointestinal system diseases**	**61**	**0.4**
Chronic digestive disorder	21	0.1
Gastrointestinal disease	40	0.2
**Others§**	**109**	**0.6**
**Grand total**	**17118**	**100**

*Patients with more than one comorbidity were missing. Calculated in column percentage format; One study (Caramelo et al. [41]) was excluded from prevalence calculation as the study had not reported frequency of comorbidities; Malnutrition and dementia was skipped from the analysis as we found only one patient (Yang et al. [10]).

†Included all types of cardiovascular diseases like coronary heart diseases or artery disease.

‡Included cerebral infarction.

§Anemia, bowel disease, tissue disease, etc.

¶Representative for this study’s included sample and could be different than the actual situation depending upon the counts in the included studies.

### Effects of pre-existing morbidity on deaths in COVID-19 patients

The pooled ORs of deaths for each category of pre-existing comorbidities among COVID-19 patients, publication bias, and Trim and Fill estimates are presented in Table 4. COVID-19 patients with pre-existing cardiovascular disease were 3.42 times more likely to die (OR = 3.42, 95% CI = 2.86-4.09; *I^2^* = 81.2%) than the patients who had no cardiovascular diseases. The odds of death among COVID-19 patients with immune and metabolic disorders were also found to be 246% higher (OR = 2.46, 95% CI = 2.03-2.85; *I^2^* = 64.8%) than among COVID-19 patients without such disorders. The incidence of COVID-19 among people with respiratory diseases increases mortality risk around two times (OR = 1.94, 95% CI = 1.72-2.19; *I^2^* = 75.7%) more than COVID-19 patients without respiratory system diseases. Similarly, we found higher mortality risk among COVID-19 patients who had pre-existing any types of cancers (OR = 2.22, 95% CI = 1.63-3.03, *I^2^* = 67.7%) and cerebrovascular system diseases (OR = 4.12, 95% CI = 3.04-5.58) more than their counterparts. Moreover, the incidence of COVID-19 among patients with pre-existing renal disease and chronic liver disease increased mortality risk by about three times (OR = 3.02, 95% CI = 2.60-3.51) more and one and half times (OR = 2.35, 95% CI = 1.50-3.69) more, respectively compared to the COVID-19 patients who did not have such comorbidities.

**Table 4:** Summary effects of type of morbidity categories on death among patients infected with COVID-19 disease, publication bias, and Trim and Fill estimates

Characteristics	Number of studies	Number of times morbidity reported	Summary estimates		Egger bias test *P*-value	Trim and fill estimates*	
**OR (95% CI)**	**Heterogeneity index (*I^2^*)**	**Missing studies No.**	**OR (95% CI)**
Cardiovascular system diseases	32	60	3.42 (2.86-4.09)†	81.2%	0.001	6	2.93 (2.46-3.49)
Immune and metabolic disorders	30	36	2.46 (2.03-2.85)†	64.8%	0.076	0	2.46 (2.03-2.85)
Respiratory system diseases	27	32	1.94 (1.72-2.19)‡	75.7%	0.053	0	1.94 (1.72-2.19)
Any types of cancer	20	20	2.22 (1.63-3.03)‡	67.7%	0.891	0	2.22 (1.63-3.03)
Cerebrovascular system diseases	15	16	4.12 (3.04-5.58)‡	25.7%	0.048	2	3.94 (2.92-5.31)
Renal system diseases	21	21	3.02 (2.60-3.51)‡	56.0%	0.024	4	2.86 (2.47-3.32)
Liver system diseases	13	16	2.35 (1.50-3.69)‡	0.0%	0.001	3	2.03 (1.33-3.11)
Gastrointestinal system diseases	5	5	1.33 (0.56-3.19)‡	0.0%	0.170	0	1.33 (0.56-3.19)

OR – odds ratio, CI – confidence interval

*The trim-and-fill method simulates studies that are likely to be missing from the literature due to publication or other forms of bias. The trim-and-fill OR estimates what the pooled OR would be if these missing studies were included in the analysis.

†Summary estimates were based on fixed-effects methods.

‡Summary estimates were based on random-effects methods. Person survived from COVID-19 infection is considered as reference category.

We found evidence of publication bias for the four categories of pre-existing comorbidities: cardiovascular diseases, cerebrovascular diseases, renal system diseases, and liver diseases (Figures S1A to S8A in the **Online Supplementary Document**). We then used Trim and Fill methods to impute the number of missing studies, which hypothetically imputed six studies for cardiovascular diseases, two studies for cerebrovascular diseases, four studies for renal diseases, and three studies for liver diseases. The pooled analysis, including these missing studies, showed almost similar results to the summary estimates presented earlier without these missing studies.

Evidence of higher deaths among COVID-19 patients with pre-existing comorbidities were also demonstrated in the narrative review (Table S5 in the **Online Supplementary Document**). In two of the three articles reviewed in this study, researchers reported that each of the patients who died following COVID-19 had pre-existing comorbidities, mostly had some type of cardiovascular diseases or immune and metabolic disorders [66,67]. Researchers in one study found around 38% of the COVID-19 patients with hypertension died [65], and one study reported higher odds for deaths in kidney injury [70].

### Stratified analysis

We found evidence of high heterogeneity (*I*^2^>75%) for cardiovascular diseases (81.2%) and respiratory diseases (75.7%). To examine the sources of heterogeneity, we conducted stratified analysis across types of comorbidities, study design (cross-sectional vs retrospective cohort vs prospective cohort), sample size (divided based on the mean sample size of the included studies and classified as ≤1134, >1134), age of the total sample (divided based on mean age and classified as ≤60 years and >60 years), and age at death (divided based on mean age and classified as ≤70 vs >70) (**Table 5** and **Table 6**). We found odds of death varied across specific types of pre-existing comorbidities included to generate the cardiovascular diseases and respiratory diseases category. For instance, in the cardiovascular diseases category, the odds of mortality were higher for COVID-19 patients with pre-existing heart failure (OR = 4.72, 95% CI = 3.19-6.97), cardiovascular disease (OR = 3.43, 95% CI = 2.52-4.66), hypertension (OR = 3.36, 95% CI = 2.64-4.28), than for COVID-19 patients with pre-existing arrthythmia (OR = 3.89, 95% CI = 2.51-6.02). Similarly, in the respiratory diseases category, the odds of mortality were found higher for COVID-19 patients with pre-existing chronic lung disease (OR = 3.34, 95% CI = 2.15-5.18), and COPD (OR = 2.62, 95% CI = 1.48-4.64).

**Table 5:** Stratified analysis of the likelihood of death among patients with cardiovascular system diseases infected with COVID-19 diseases

Characteristics	Pooled OR (95% CI)	*P*-value	
**Heterogeneity**	**Meta-regression**
**Type of diseases:**			
Cardiovascular diseases	3.43 (2.52-4.66)	<0.01	0.93
Hypertension	3.36 (2.64-4.28)	<0.01	
Heart failure	4.72 (3.19-6.97)	0.643	
Arrhythmia	3.89 (2.51-6.02)++	NA	
**Study country:**			
Australia	3.68 (2.06-6.57)++	NA	<0.01
Italy	3.82 (2.90-5.02)	0.368	
China	4.24 (3.30-5.44)	<0.01	
United State of America	2.74 (2.00-3.75)	<0.01	
Iran	1.75 (0.95-3.22)	0.685	
UK	2.73 (1.17-6.35)++	NA	
Mexico	1.12 (0.71-1.75)	<0.05	
**Study design:**			
Cross-sectional	1.12 (0.71-1.75)	<0.05	<0.01
Prospective cohort	3.70 (2.67-5.12)	<0.01	
Retrospective cohort	3.58 (2.93-4.37)	<0.01	
**Adjustment factor:**			
Unadjusted	2.79 (1.60-4.84)	<0.01	<0.05
Adjusted	1.53 (0.96-2.46)	<0.01	
**Sample size:**			
>1134	2.33 (1.53-3.55)	<0.01	0.091
≤1134	3.70 (3.07-4.45)	<0.01	
**Mean age of the total sample:**			
≤60	4.46 (3.00-6.64)	<0.01	0.593
>60	3.00 (2.48-3.62)	<0.01	
**Mean age of death sample:**			
≤70	5.13 (3.92-7.20)	<0.01	<0.01
>70	2.54 (2.06-3.12)	<0.01	

**Table 6:** Stratified analysis of the likelihood of death among patients with respiratory system diseases infected with COVID-19 diseases

Characteristics	Pooled OR (95% CI)	*P*-value	
**Heterogeneity**	**Meta-regression**
**Type of diseases:**			
Chronic lung disease	3.34 (2.15-5.18)	<0.01	0.232
Chronic obstructive pulmonary disease (COPD)	2.62 (1.48-4.64)	<0.01	
Pulmonary emphysema	4.69 (0.41-52.97) ++	NA	
Chronic bronchitis	1.53 (0.41-5.68)++	NA	
Asthma	1.01 (0.61-1.67)	0.152	
**Study country:**			
Australia	4.56 (2.47-8.44)++	NA	0.214
Italy	1.54 (0.73-3.25)	**NA**	
China	3.46 (2.20-5.43)	<0.01	
United State of America	2.11 (1.26-3.52)	<0.01	
Iran	2.16 (1.05-4.45) ++	NA	
Mexico	1.20 (0.54-2.68)	<0.01	
**Study design:**			
Cross-sectional	1.20 (0.54-2.68)	<0.05	0.221
Prospective cohort	2.76 (1.45-5.23)	<0.01	
Retrospective cohort	2.98 (2.00-4.44)	<0.01	
**Adjustment factor:**			
Unadjusted	2.79 (1.60-4.84)	<0.01	<0.05
Adjusted	1.53 (0.96-2.46)	<0.01	
**Sample size:**			
>1134	1.88 (1.14-3.09)	<0.01	0.161
≤1134	2.97 (2.11-4.16)	<0.01	
**Mean age of the total sample:**			
≤60	3.41 (1.86-6.26)	<0.01	0.279
>60	2.32 (2.63-3.29)	<0.01	
**Mean age of death sample:**			
≤70	4.22 (2.44-7.30)	0.209	0.055
>70	2.16 (1.57-2.99)	<0.01	

## DISCUSSION

This study aimed to summarize pre-existing comorbidities among COVID-19 patients, which increases their incidence of deaths and their corresponding likelihoods. A total of 42 studies were included that comprised 27 670 samples, and 42.5% of them had any pre-existing comorbidities. The most frequently reported comorbidities were hypertension (39.5%), diabetes (25.2%), and cardiovascular disease (12.4%). The likelihood of death was higher among COVID-19 patients who had comorbidities like cardiovascular diseases, cerebrovascular diseases, respiratory diseases, renal diseases, immune and metabolic disorders, hepatic diseases, and cancer. This evidence will guide physicians to take precautionary measures, which could reduce the number of fatalities following a secondary infection with COVID-19.

Among the total positive COVID-19 cases included in this systematic review, around 43% had one or more pre-existing comorbidities, mostly cardiovascular diseases, and immune and metabolic disorders. These underlying diseases are increasing the risk of death among COVID-19 patients. Recent reviews have confirmed that COVID-19 infection among patients with cardiovascular diseases, and immune and metabolic disorders, particularly, having hypertension and diabetes elevate the risk of infection severity, ICU admission, and the risk of death [19,23]. The underlying causes are that patients with these diseases are more likely to have a higher neutrophil-lymphocyte ratio [71,72], higher D-dimer level [73], and higher C-reactive protein [74]. These are responsible for multiple organ failure [75,76], severe pneumonia, hypoxia, respiratory failure, myocardial damage, and circulatory failure [77], and these risks increase further if the patient is infected with COVID-19 [21,44,76,78] because of additional damage of myocardial cells [79,80]. Importantly, a similar higher risk of mortality was reported among the patients of cardiovascular diseases infected with the SARS-CoV and MERS-CoV [81,82]. These two diseases are considered as ancestors of the current COVID-19, which were reported in 2003 and 2012, respectively [44,81-83]. Evidence also validates that the occurrence of other viral infections like influenza, similar to COVID-19, could increase the risk of death if the patient has underlying cardiovascular diseases and diabetes [84].

Pooled likelihoods in this study provide evidence of higher deaths among COVID-19 patients who had pre-existing chronic respiratory diseases or any type of cancer- as found in other reviews [19,23]. The chronic respiratory diseases like COPD and asthma are well-established risk factors for pneumonia [85], which also increase the susceptibility to COVID-19 infection [86]. Once patients are infected with COVID-19, these underlying diseases further affect the patient’s respiratory system and progress to severe hypoxemia [87]; therefore, the cumulative effects lead to the events of death [77,86]. Cancer patients are more likely to report a systemic immunosuppressive state and progress to severe clinical events in COVID-19, such as require intensive care (ICU) or death [88,89]. These underlying diseases have their own adverse consequences on the human body. Secondary infection of COVID-19, which could, therefore, increase serve clinical events as well as deaths among patients with these types of pre-existing comorbidities.

This study also suggests that patients with cerebrovascular, liver, and renal diseases are more vulnerable to mortality following the secondary incidence of COVID-19 than patients who do not have such diseases. The results are comparable to deaths among previously reported SARS patients [90]. Diseases such as cardio-cerebrovascular diseases, liver damage, or renal diseases accelerate an abrupt loss of kidney function [91,92], and tissue damage that causes hypoxia, shock, and rhabdomyolysis [70,93]. Importantly, these states also increase the occurrence of thrombocytopenia (reduced platelet counts in the blood) [21,94]. Together with COVID-19, these adverse effects could potentially elevate the risk of progression to a severe state or death of a COVID-19 patient. Moreover, elevated alanine aminotransferase (ALT) levels and reduced albumin levels are found to be associated with higher mortality in COVID-19 [21,94], which can be caused by chronic liver and kidney diseases [94-98].

### Strengths and limitations

This study has several strengths and limitations that should be reported. To our knowledge, this is the first study of its kind that summarizes all morbidities among COVID-19 patients that lead to death using data from globally representative studies. Moreover, comorbidities reported among COVID-19 patients were classified into board groups based on their characteristics, and the likelihood of death was estimated separately for each group. This evidence will inform health care providers about the risk of death among COVID-19 patients with different groups of pre-existing comorbidities. Thus, they will be able to take precautionary measures early targeting to prevent deaths. However, this study reported the odds of death for COVID-19 patients with one pre-existing comorbidity only. Many COVID-19 patients may have multi-morbidities (COVID-19 with pre-existing two or more comorbidities), and they may have a higher risk of death. The studies included in this review considered each morbidity separately; for instance, if COVID-19 patients had both hypertension and diabetes, they were included in both groups. None of the included studies considered COVID-19 patients with two or more comorbidities together; therefore, we failed to provide the likelihood of deaths for higher risk patients. Moreover, the percentage distribution of morbidity among COVID-19 patients estimated in this study could be different from the actual situation depending upon the counts in the included studies. However, as we included all available studies conducted worldwide, therefore, any distortion is likely to be random. In addition, the likelihoods presented in this study were mostly unadjusted (31 of the 35 articles included) as they were calculated from the extracted raw data. This may overestimate or underestimate the actual likelihood of deaths in COVID-19 patients because age and other socio-demographic characteristics are potential confounders of their deaths, which should be adjusted for unbiased estimates. Despite these limitations, this study is unique and beneficial for health care providers to handle COVID-19 patients with pre-existing comorbidities.

## CONCLUSION

About 43% of the sample included in this systematic review had one or more pre-existing comorbidities and had COVID-19 as a secondary infection. The most common pre-existing comorbidities were hypertension, diabetes, and cardiovascular disease. The likelihood of death was higher among COVID-19 patients who had pre-existing cardiovascular and cerebrovascular diseases, respiratory diseases, renal diseases, immune and metabolic disorders, liver diseases, and any types of cancer. These findings will help health care providers to sort COVID-19 patients by comorbidities, take precautionary measures during hospitalization, assess susceptibility to death, and prioritize their treatment. These could potentially reduce the number of fatalities from secondary infection with COVID-19 disease.

## Additional material

Online Supplementary Document
