# Supplementary material for: Effects of underlying morbidities on the occurrence of deaths in COVID-19 patients: A systematic review and meta-analysis
Source: J Glob Health. 2020 Aug 18;10(2):020503. doi: 10.7189/jogh.10.020503 (PMC7567434; doi:10.7189/jogh.10.020503)
Supplement: Online Supplementary Document [file jogh-10-020503-s001.pdf]

Effects of underlying morbidities on the occurrence of deaths in COVID-19 patients: A systematic review and meta-analysis

Supplemental Tables

Table S1. Medline search results for pre-existing morbidities among COVID-19 patients

#	Searches	Results
1	(COVID-19 or 2019-nCoV or Coronavirus or SARS-CoV-2).mp. [mp=title, abstract, original title, name of substance word, subject heading word, floating sub-heading word, keyword heading word, organism supplementary concept word, protocol supplementary concept word, rare disease supplementary concept word, unique identifier, synonyms]	17285
2	(Comorbidit* or Morbidit*).mp. [mp=title, abstract, original title, name of substance word, subject heading word, floating sub-heading word, keyword heading word, organism supplementary concept word, protocol supplementary concept word, rare disease supplementary concept word, unique identifier, synonyms]	573824
3	(Mortalit* or Death or Died*).mp. [mp=title, abstract, original title, name of substance word, subject heading word, floating sub-heading word, keyword heading word, organism supplementary concept word, protocol supplementary concept word, rare disease supplementary concept word, unique identifier, synonyms]	1826650
4	1 and 2 and 3	433
5	limit 4 to (english language and yr="2019 -Current")	146

Table S2. CINAHL search results for pre-existing morbidities among COVID-19 patients

#	Query	Limiters/Expanders	Last Run Via	Results
S1	“"(covid-19 OR 2019-ncov OR coronavirus OR sars-cov-2) AND (comorbidit* OR morbidit*) AND (mortalit* OR deat* OR died)	Limiters - Published Date: 20191201-20200431; English Language<br><br>Expanders - Apply equivalent subjects<br><br>Search modes - Boolean/Phrase	Interface - EBSCOhost Research Databases<br>Search Screen - Advanced Search<br>Database - CINAHL Complete	19

Table S3. Web of science search results for pre-existing morbidities among COVID-19 patients

Search terms	Results
(covid-19 OR 2019-ncov OR coronavirus OR sars-cov-2) *AND* TOPIC: (comorbidit* OR morbidit*) *AND* TOPIC: (mortalit* OR deat* OR died)<br>**Timespan:** 2019-2020. **Indexes:** SCI-EXPANDED, SSCI, A&HCI, CPCI-S, CPCI-SSH, ESCI, CCR-EXPANDED, IC	64

Table S4. SCOPUS search results for pre-existing comorbidities and the mortality risk in COVID-19

Search strategy	Results
TITLE-ABS-KEY (((covid-19 OR 2019-ncov OR coronavirus OR sars-cov-2) AND (comorbidit* OR morbidit*) AND (mortalit* OR death* OR died))) AND (LIMIT-TO (PUBYEAR , 2020) OR LIMIT-TO (PUBYEAR , 2019))	142

Table S5. Narrative review for pre-existing morbidities and mortality risk among patients in COVID-19 infection.

Study	Study design, Country	Sample	Results
Grasselli et al., 2020	Retrospective cohort, Italy	Total of 1591 COVID-19 patients admitted into ICU in 72 hospitals from 20 February to 18 March 2020	Around 49% of the patients had hypertension, 21% had cardiovascular disease, 18% had hypercholesterolemia, 17% had type-2 diabetes, and 8% had malignancy. A total of 405 (26%) patients died. Around 38% of the patients with hypertension were died in ICU as compared to 16% discharged.
Du et al., 2020	Prospective cohort, China	Total of 109 died COVID-19 patients from three hospitals in Wuhan, China. Data were collected from 25 Dec to 15 February 2020	COVID-19 patients who were died mostly had pre-existing hypertension (59.6%), cardiovascular disease (33.9%), diabetes (31.2%), digestive disorders (16%), chronic respiratory diseases (15.6%), malignancy (7.3%), chronic kidney disease (7.3%), 7.3% had peripheral vascular disease.
Zhang et al., 2020	Retrospective cohort, China	Total of 82 died COVID-19 patients admitted Wuhan University's hospital from 11 January to 10 February 2020.	Patients died following secondary COVID-19 mostly had pre-existing hypertension (56.1%) following cardiovascular disease (20.7%), diabetes (18.3%), immunodeficiency (17.1%) chronic respiratory diseases (14.6%), cerebrovascular disease (12.2%), malignancy (7.3%), chronic kidney disease (4.9%), and chronic liver disease (2.4%).
Kim et al., 2020	Retrospective cohort, Korea	Sample consisting of 101 deceased patients from February 19 to March 20, 2020	COVID-19 patients who were died mostly had pre-existing hypertension (64.4%), cardiovascular disease (21.8%), diabetes (43.6%), digestive disorders (16%), chronic respiratory diseases (27.7%), dementia (25.7), dyslipidaemia (15.8%), cerebrovascular disease (15.8%), malignancy (15.8%), and renal diseases (14.8%).
Yao et al., 2020	Retrospective cohort, China	Sample of 55 patients who died were collected from East Hospital of Wuhan University as of February 18, 2020	Patients died following secondary COVID-19 mostly had pre-existing hypertension (60%) diabetes (26%), cardiovascular (31%), cerebrovascular disease (22%), malignancy (7%), chronic lung diseases (22%), chronic kidney disease (9%), and chronic liver disease (6%).
Cheng et al., 2020	Prospective cohort, China	Total of 701 COVID-19 patients from China	Around 16.1% (113 person) died in hospital. Patients with acute kidney injury were reported higher risk of death with a gradual increase across stages of injury: stage 1 (HR, 3.51; 95% CI, 1.53-8.02), stage 2 (HR, 6.24; 95% CI, 2.73-14.27), stage 3 (HR, 9.81; 95% CI, 5.46-17.65).

Table S6. Newcastle-Ottawa scale assessment of study quality for **cross-sectional study**

Author	Selection				Comparability	Outcome		Study quality
	1	2	3	4	5	6	7	
	Representativeness of the sample	Sample size	Ascertainment of exposure	Non-respondents	The subjects in different outcome groups are comparable, based on the study design or analysis. Confounding factors are controlled.	Assessment of outcome	Statistical test is appropriate	
Solis et al., 2020	*	*	*		*		*	5

Table S7. Newcastle-Ottawa scale assessment of study quality for **cohort study**

Author	Selection				Comparability		Outcome			Study quality
	1	2	3	4	5A	5B	6	7	8	
	Exposed cohort truly representative	Non-exposed cohort drawn from the same community	Ascertai n-ment of exposure	Outcome of interest not present at start	Cohorts comparable on basis of age	Cohorts comparable on other factor(s)	Quality of outcome assessment	Follow-up long enough for outcomes to occur	Complete accounting for cohorts	
Guan et al., 2020	*	*	*		*	*	*	*	*	8
Cao et al., 2020	*	*	*		*	*	*	*	*	8
Chen et al., 2020	*	*	*		*	*	*	*	*	8
Deng et al., 2020	*	*	*			*	*	*	*	7

Yang et al., 2020	*	*	*			*	*		*	*	*	8
Wu et al., 2020		*	*				*		*	*	*	6
Chen et al., 2020	*	*	*			*	*		*	*	*	8
Zhou et al., 2020	*	*	*			*	*		*	*	*	8
Yuan et al., 2020	*	*	*				*		*	*	*	7
Chen et al., 2020	*		*	*			*		*	*		6
Caramelo et al., 2020	*					*	*			*	*	5
Ren et al., 2020	*	*	*			*	*		*	*	*	8
Australian Government., 2020	*	*				*	*		*	*	*	7
Shi et al., 2020	*		*			*	*			*	*	6
Zhang et al., 2020	*	*		*		*	*		*	*		7
Du et al., 2020	*	*	*			*	*		*	*	*	8
Wang et al., 2020	*	*	*	*		*	*			*	*	8
Fu et al., 2020	*	*				*	*			*	*	6
Paranjpe et al., 2020	*	*	*				*		*	*		6
Cummings et al., 2020	*	*	*			*	*		*	*		7
Guo et al., 2020		*	*	*			*			*	*	6
Zhu et al., 2020	*			*		*	*		*	*		6
Yin et al., 2020		*	*	*			*		*	*	*	7
Sun et al., 2020		*	*	*					*	*		5

Luo et al., 2020	*	*	*			*	*		*	*	*	8
Zhang et al., 2020	*	*	*	*			*		*	*	*	8
Yao et al., 2020		*	*	*		*	*		*	*		7
Zangrillo et al., 2020		*	*	*		*	*		*	*		7
Yan et al., 2020		*	*	*		*	*		*			6
Tedeschi et al., 2020		*	*	*		*				*	*	6
Nikpouraghdam et al., 2020	*	*	*	*		*	*			*	*	8
Benelli et al., 2020	*	*	*			*	*		*	*		7
Levy et al., 2020	*	*	*	*		*	*			*	*	8
Sneep et al., 2020		*	*			*			*	*		5
Mehra et al., 2020	*	*	*	*		*	*		*	*	*	9
Grasselli et al., 2020	*		*	*			*		*	*		6
Du et al., 2020	*		*	*		*	*		*	*		7
Zhang et al., 2020	*		*	*		*	*		*	*		7
Kim et al., 2020	*		*			*	*		*	*		6
Yao et al., 2020	*		*			*	*		*	*		6
Cheng et al., 2020	*		*				*		*	*	*	6

Section/topic	#	Checklist item	Reported on page #
**TITLE**			
Title	1	Identify the report as a systematic review, meta-analysis, or both.	1
**ABSTRACT**			
Structured summary	2	Provide a structured summary including, as applicable: background; objectives; data sources; study eligibility criteria, participants, and interventions; study appraisal and synthesis methods; results; limitations; conclusions and implications of key findings; systematic review registration number.	1-2
**INTRODUCTION**			
Rationale	3	Describe the rationale for the review in the context of what is already known.	3
Objectives	4	Provide an explicit statement of questions being addressed with reference to participants, interventions, comparisons, outcomes, and study design (PICOS).	4
**METHODS**			
Protocol and registration	5	Indicate if a review protocol exists, if and where it can be accessed (e.g., Web address), and, if available, provide registration information including registration number.	NA
Eligibility criteria	6	Specify study characteristics (e.g., PICOS, length of follow-up) and report characteristics (e.g., years considered, language, publication status) used as criteria for eligibility, giving rationale.	5
Information sources	7	Describe all information sources (e.g., databases with dates of coverage, contact with study authors to identify additional studies) in the search and date last searched.	4
Search	8	Present full electronic search strategy for at least one database, including any limits used, such that it could be repeated.	4, Supplementary table 1-4
Study selection	9	State the process for selecting studies (i.e., screening, eligibility, included in systematic review, and, if applicable, included in the meta-analysis).	5
Data collection process	10	Describe method of data extraction from reports (e.g., piloted forms, independently, in duplicate) and any processes for obtaining and confirming data from investigators.	5

Data items	11	List and define all variables for which data were sought (e.g., PICOS, funding sources) and any assumptions and simplifications made.	5
Risk of bias in individual studies	12	Describe methods used for assessing risk of bias of individual studies (including specification of whether this was done at the study or outcome level), and how this information is to be used in any data synthesis.	NA
Summary measures	13	State the principal summary measures (e.g., risk ratio, difference in means).	7
Synthesis of results	14	Describe the methods of handling data and combining results of studies, if done, including measures of consistency (e.g., I^2) for each meta-analysis.	7

Section/topic	#	Checklist item	Reported on page #
Risk of bias across studies	15	Specify any assessment of risk of bias that may affect the cumulative evidence (e.g., publication bias, selective reporting within studies).	NA
Additional analyses	16	Describe methods of additional analyses (e.g., sensitivity or subgroup analyses, meta-regression), if done, indicating which were pre-specified.	6
**RESULTS**			
Study selection	17	Give numbers of studies screened, assessed for eligibility, and included in the review, with reasons for exclusions at each stage, ideally with a flow diagram.	7
Study characteristics	18	For each study, present characteristics for which data were extracted (e.g., study size, PICOS, follow-up period) and provide the citations.	7
Risk of bias within studies	19	Present data on risk of bias of each study and, if available, any outcome level assessment (see item 12).	NA
Results of individual studies	20	For all outcomes considered (benefits or harms), present, for each study: (a) simple summary data for each intervention group (b) effect estimates and confidence intervals, ideally with a forest plot.	8, Tables 1, and

			Supplementary table 5
Synthesis of results	21	Present results of each meta-analysis done, including confidence intervals and measures of consistency.	8-9
Risk of bias across studies	22	Present results of any assessment of risk of bias across studies (see Item 15).	9
Additional analysis	23	Give results of additional analyses, if done (e.g., sensitivity or subgroup analyses, meta-regression [see Item 16]).	9-10
**DISCUSSION**			
Summary of evidence	24	Summarize the main findings including the strength of evidence for each main outcome; consider their relevance to key groups (e.g., healthcare providers, users, and policy makers).	10
Limitations	25	Discuss limitations at study and outcome level (e.g., risk of bias), and at review-level (e.g., incomplete retrieval of identified research, reporting bias).	12
Conclusions	26	Provide a general interpretation of the results in the context of other evidence, and implications for future research.	13
**FUNDING**			
Funding	27	Describe sources of funding for the systematic review and other support (e.g., supply of data); role of funders for the systematic review.	Title page

From: Moher D, Liberati A, Tetzlaff J, Altman DG, The PRISMA Group (2009). Preferred Reporting Items for Systematic Reviews and Meta-Analyses: The PRISMA Statement. PLoS Med 6(6): e1000097. doi:10.1371/journal.pmed1000097

Supplementary figures

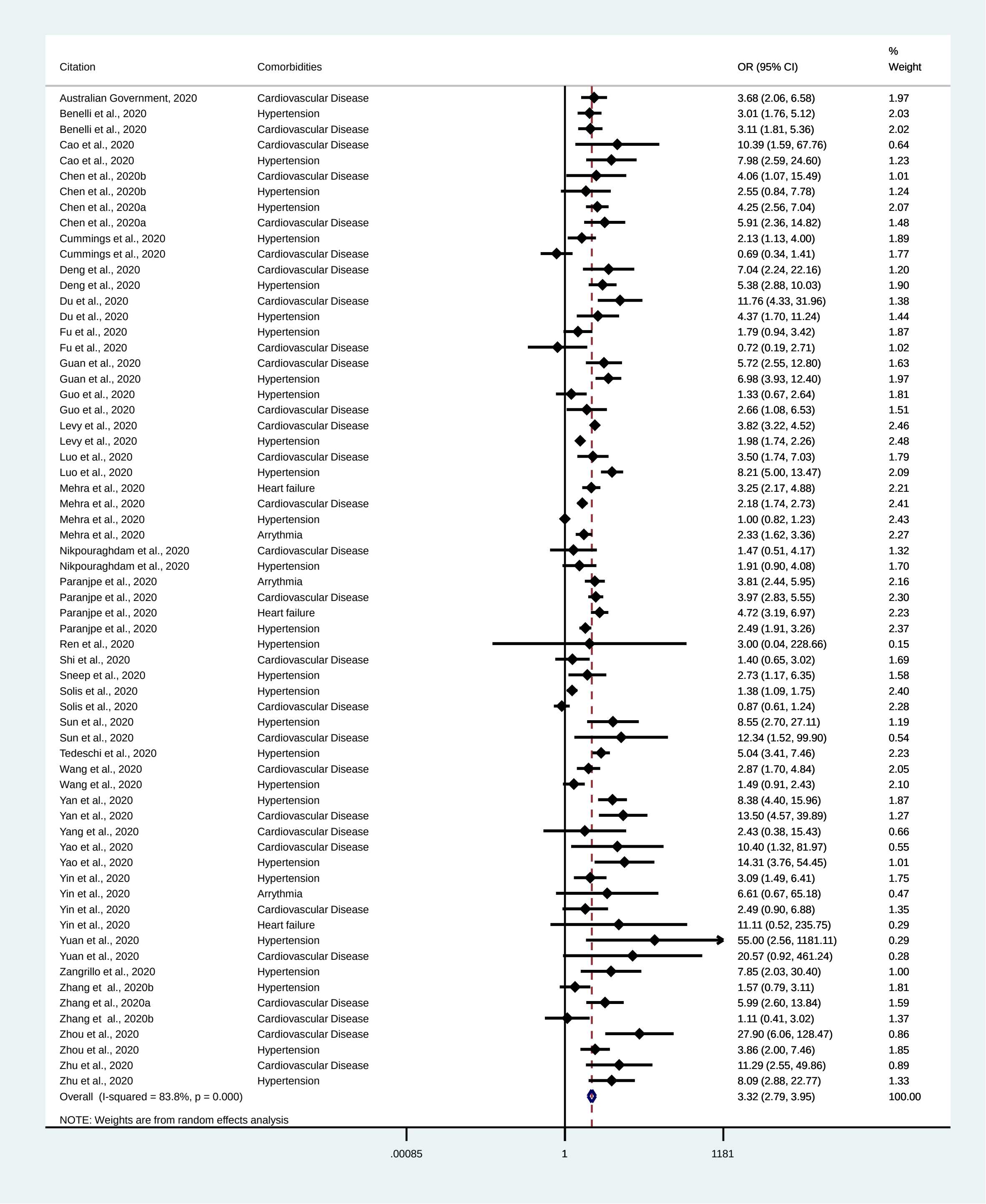

Figure S1. Likelihoods of death among patients with cardiovascular system diseases infected further with COVID-19 disease

(a)

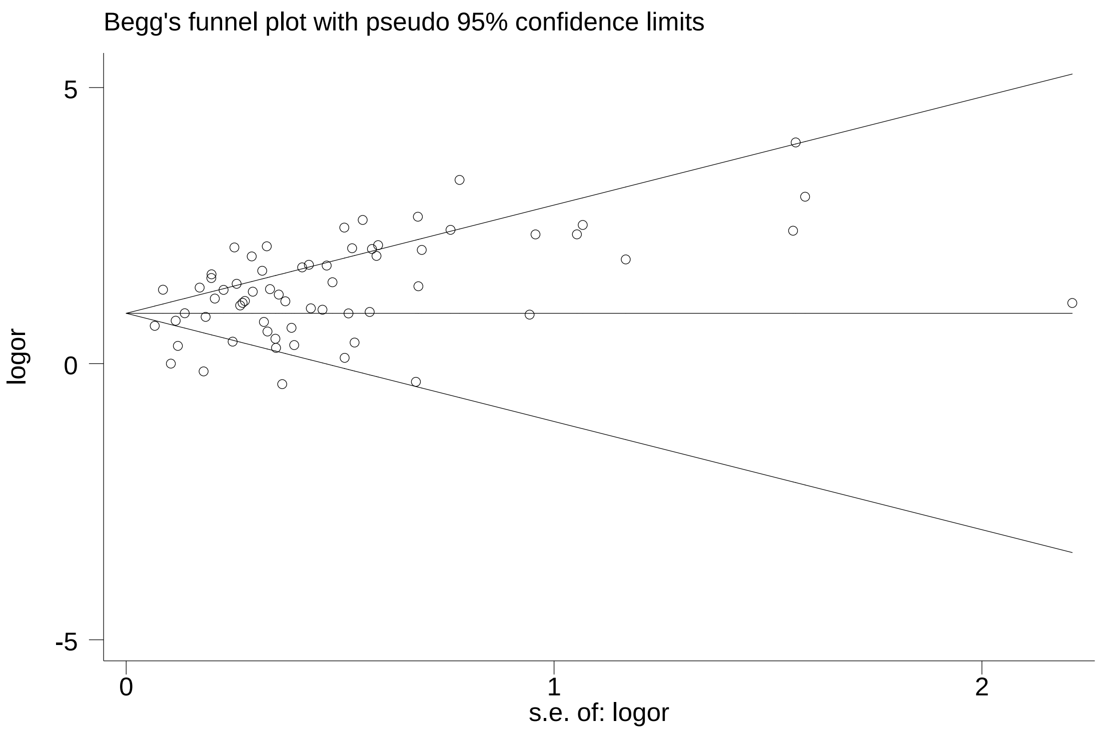

(b)

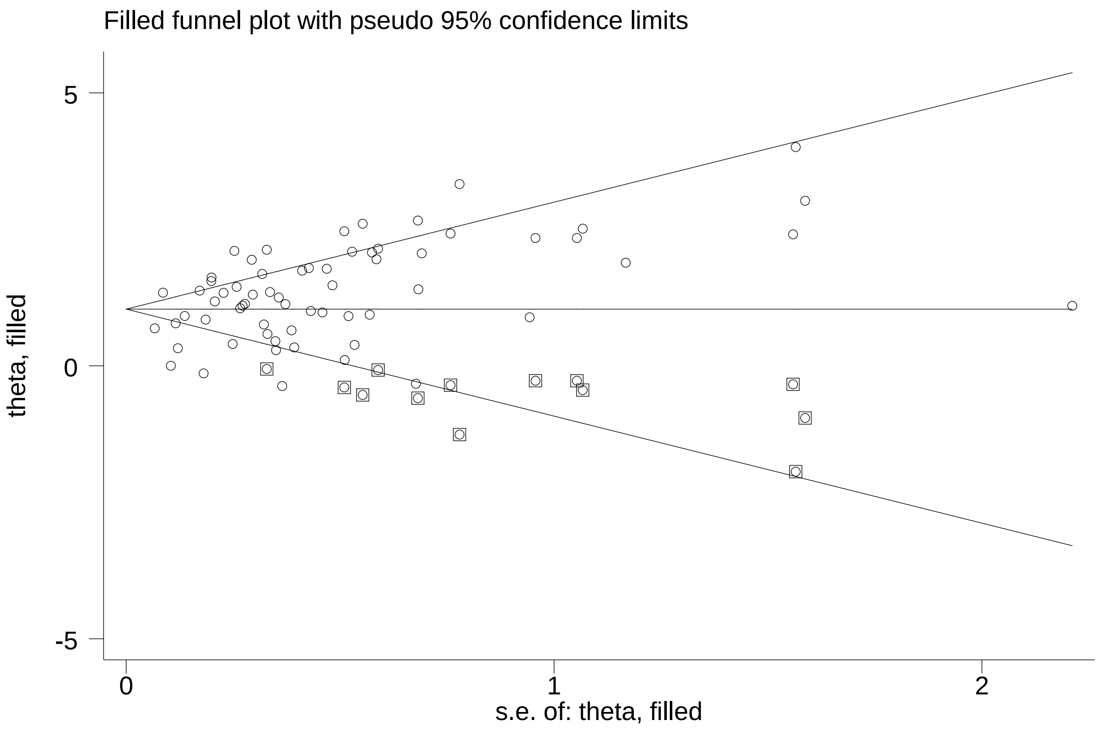

Figure S1a. Funnel plot without (a) and with trim and fill (b) estimate for cardiovascular systems diseases patients infected further with COVID-19 disease

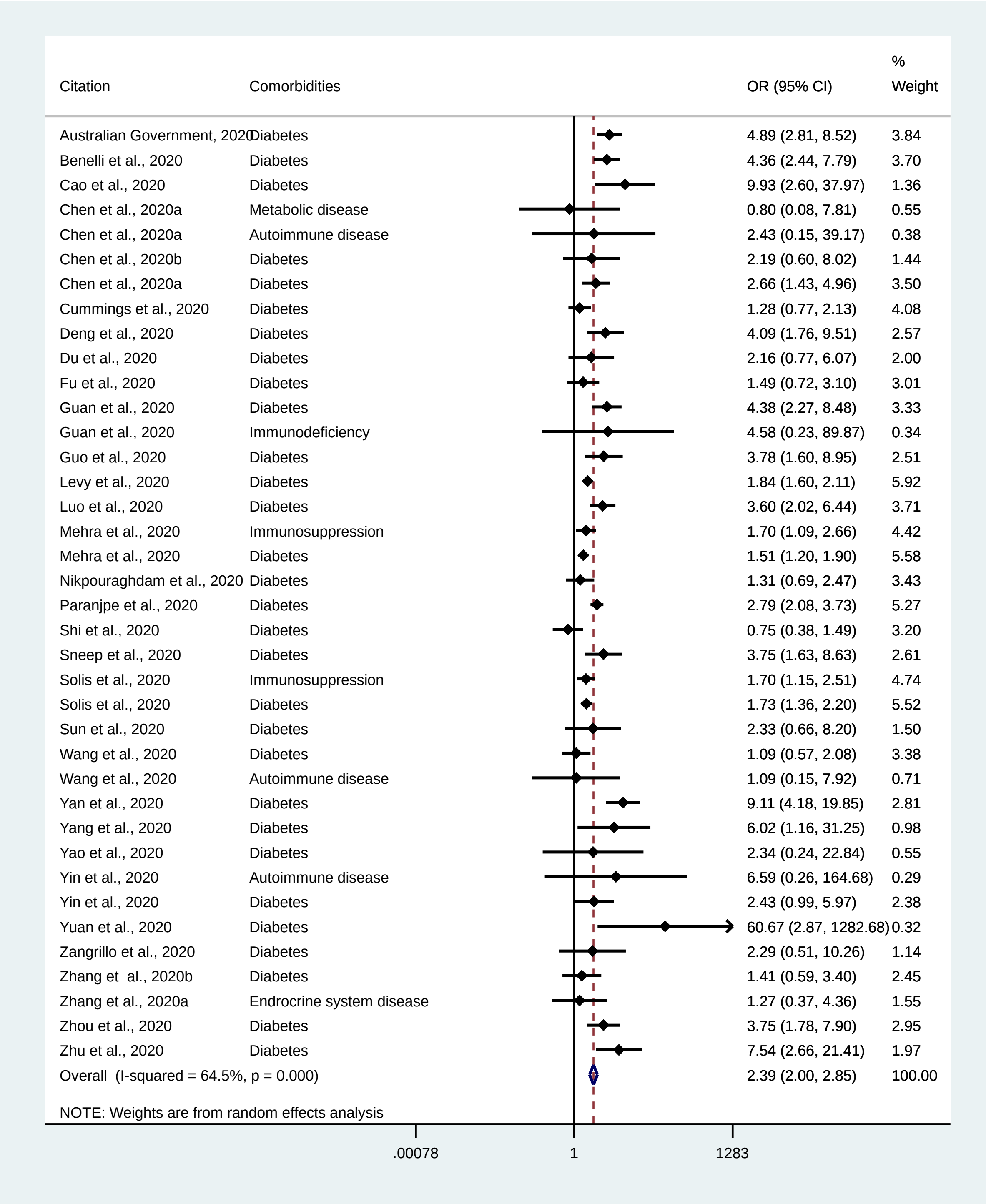

Figure S2. Likelihoods of death among patients with Immune and metabolic disorders patients infected further with COVID-19 disease

(a)

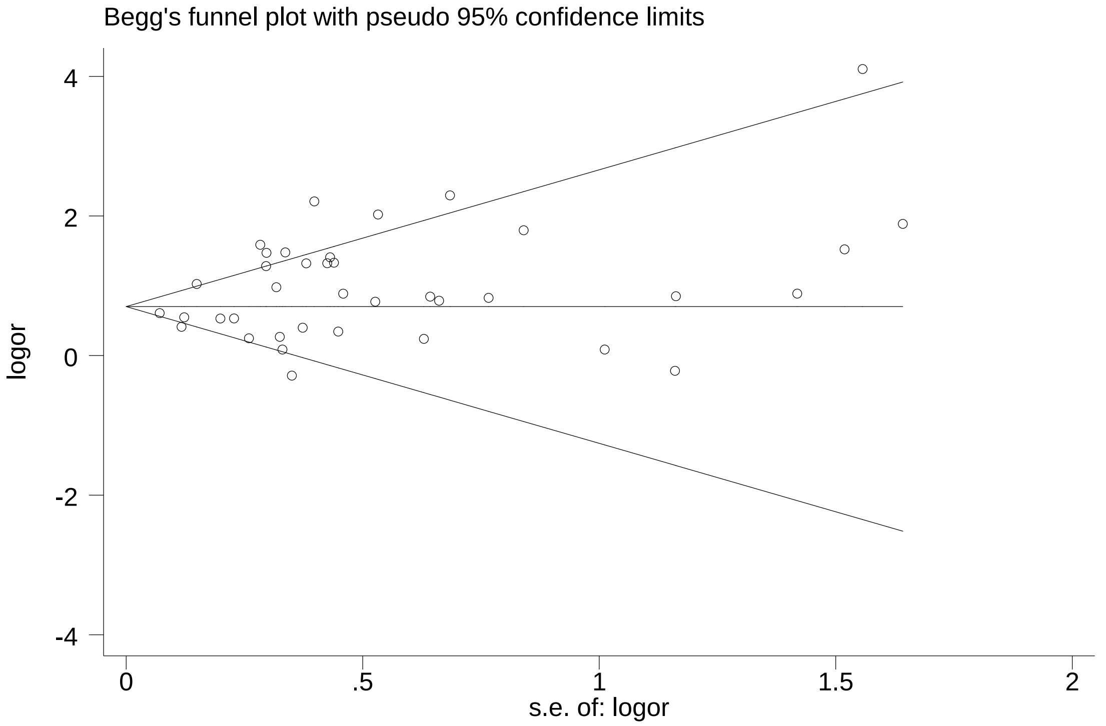

(b)

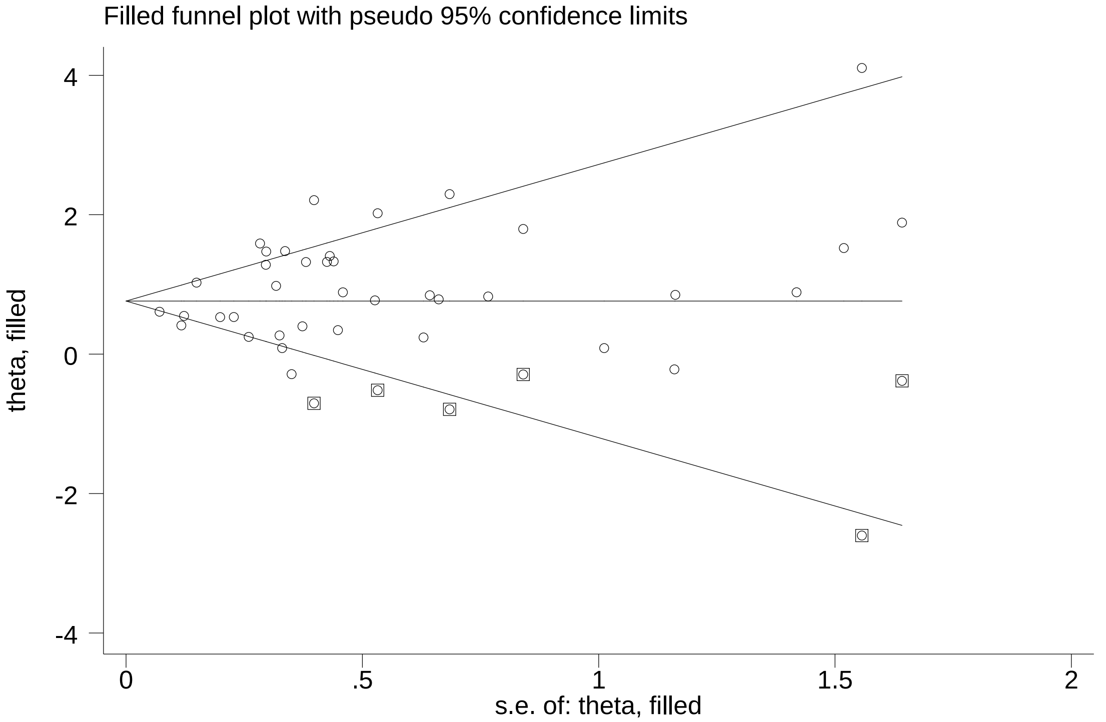

Figure S2a. Funnel plot without (a) and with trim and fill (b) estimate for Immune and metabolic disorders patients infected further with COVID-19 disease

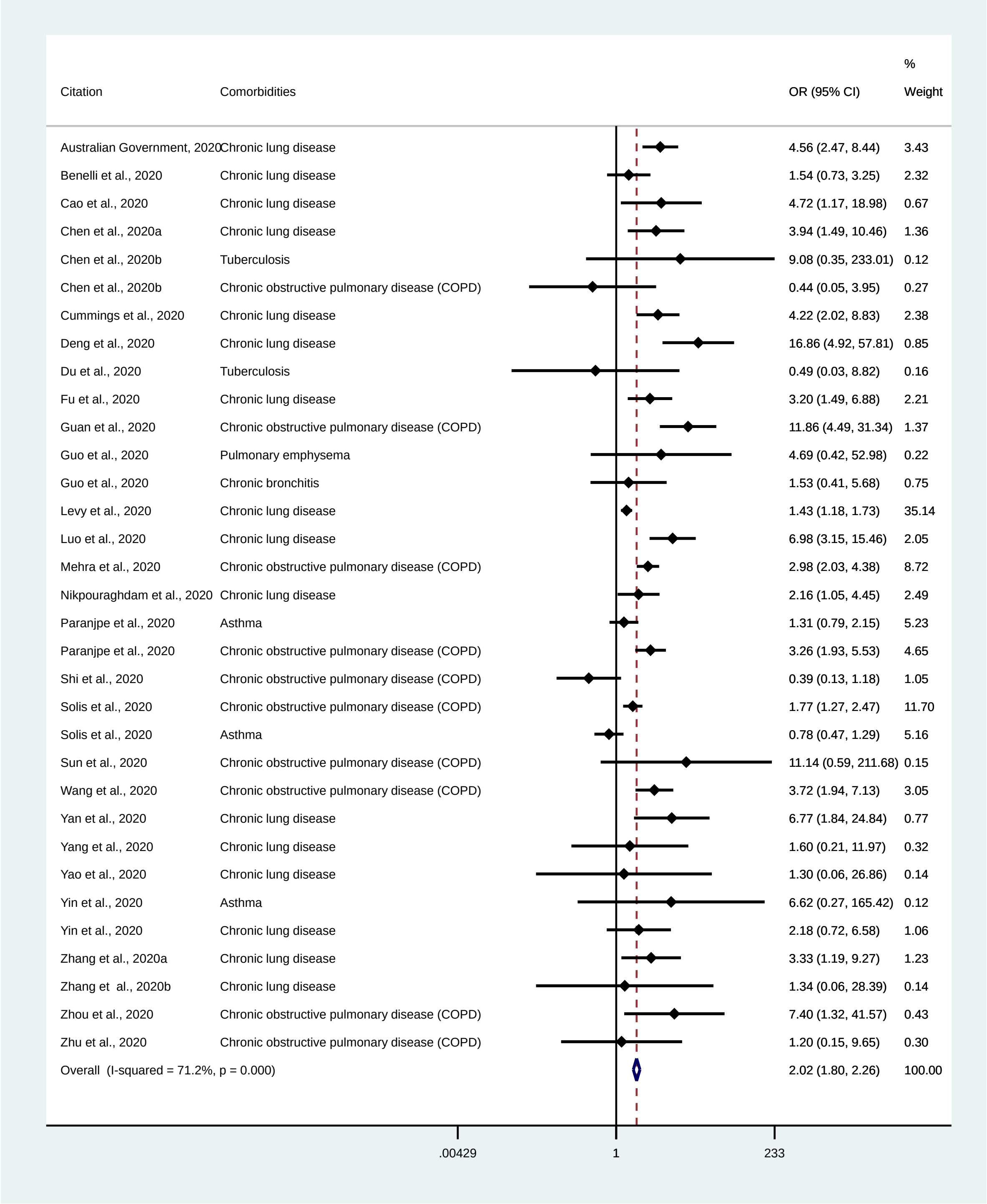

Figure S3. Likelihoods of death among patients with respiratory system diseases patients infected further with COVID-19 disease

(a)

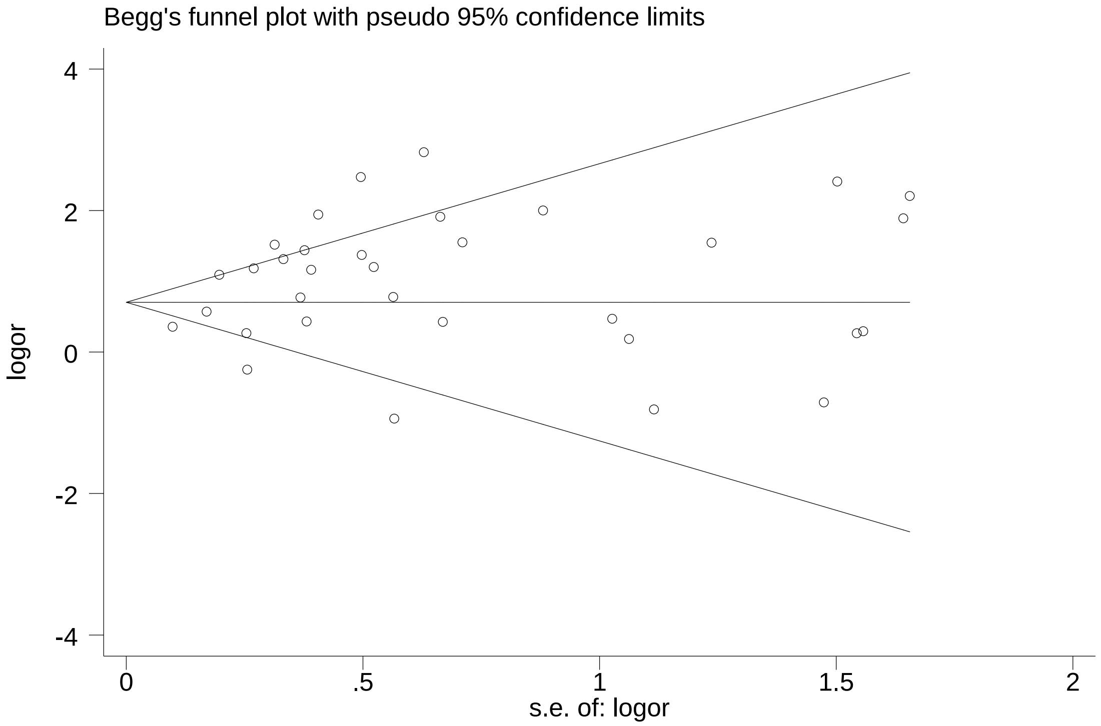

(b)

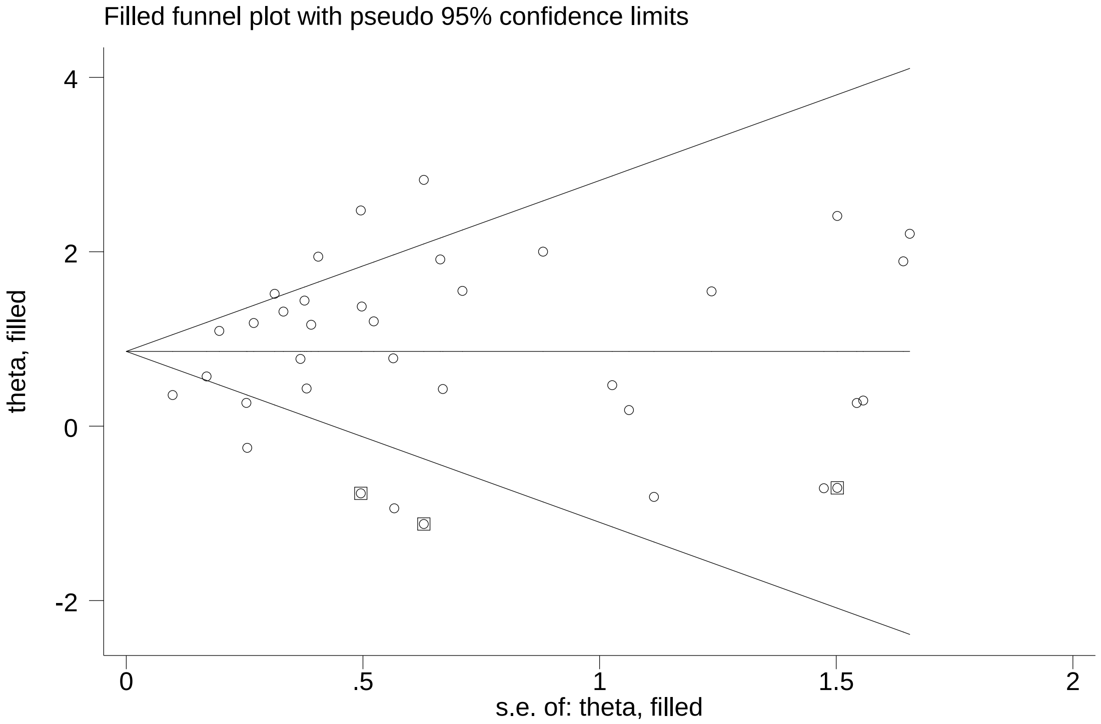

Figure S3a. Funnel plot without (a) and with trim and fill (b) estimate for Respiratory system diseases patients infected further with COVID-19 disease

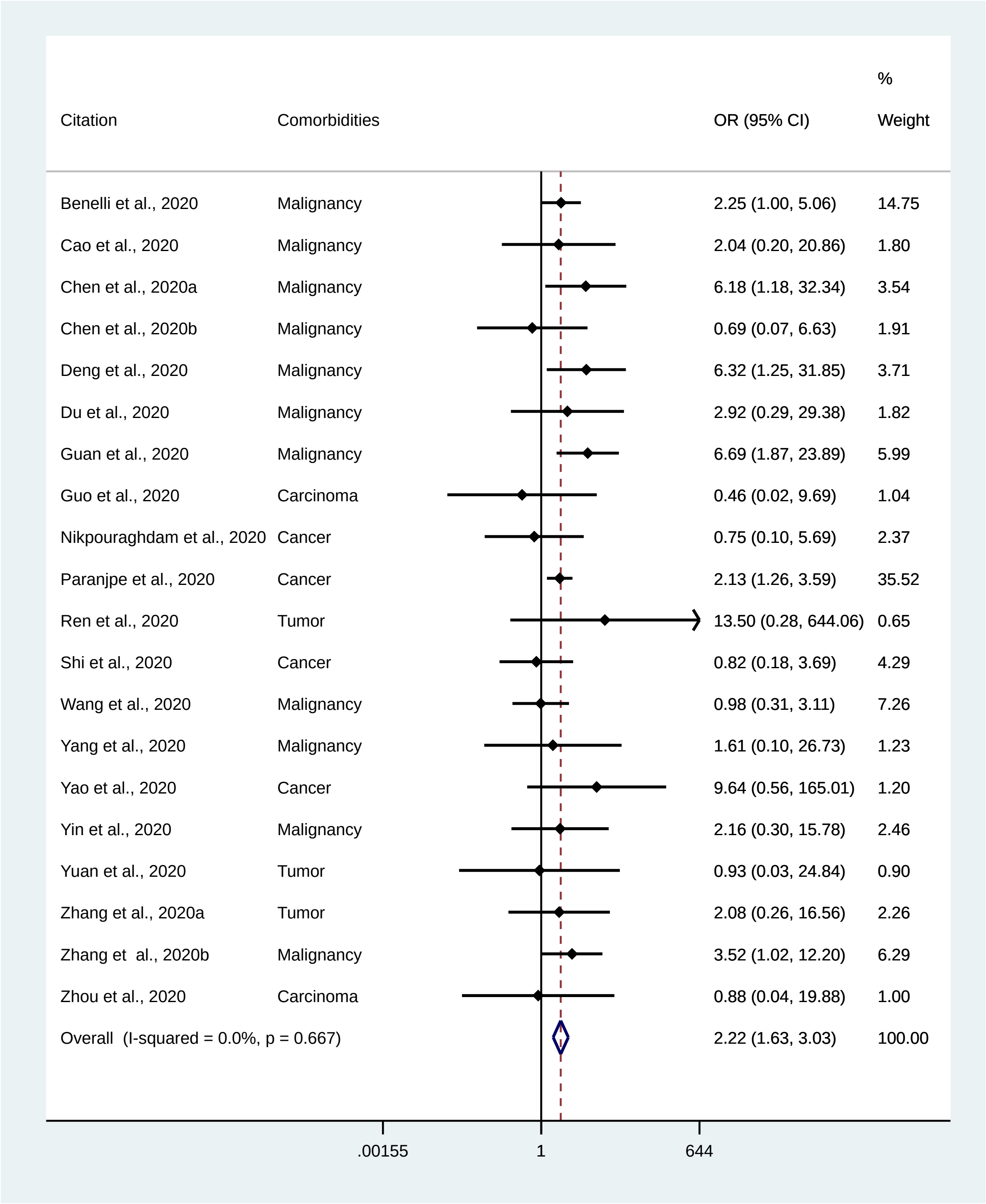

Figure S4. Likelihoods of death among patients with any type of cancers infected further with COVID-19 disease

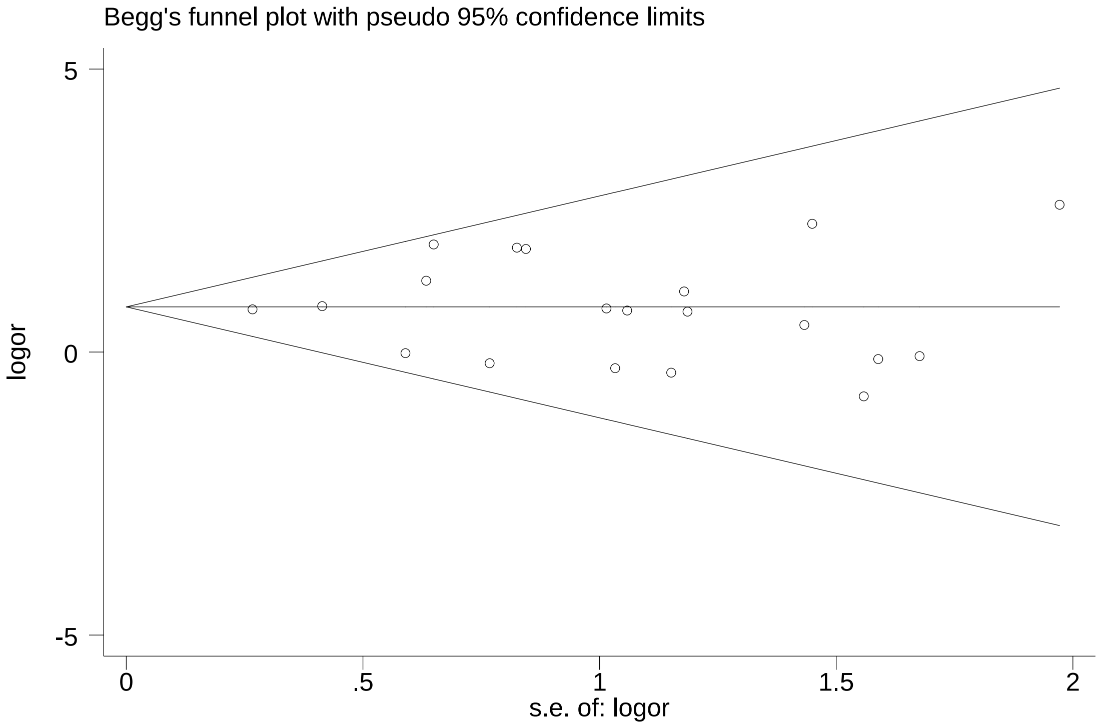

Figure S4a. Funnel plot for patients with any type of cancers infected further with COVID-19 disease

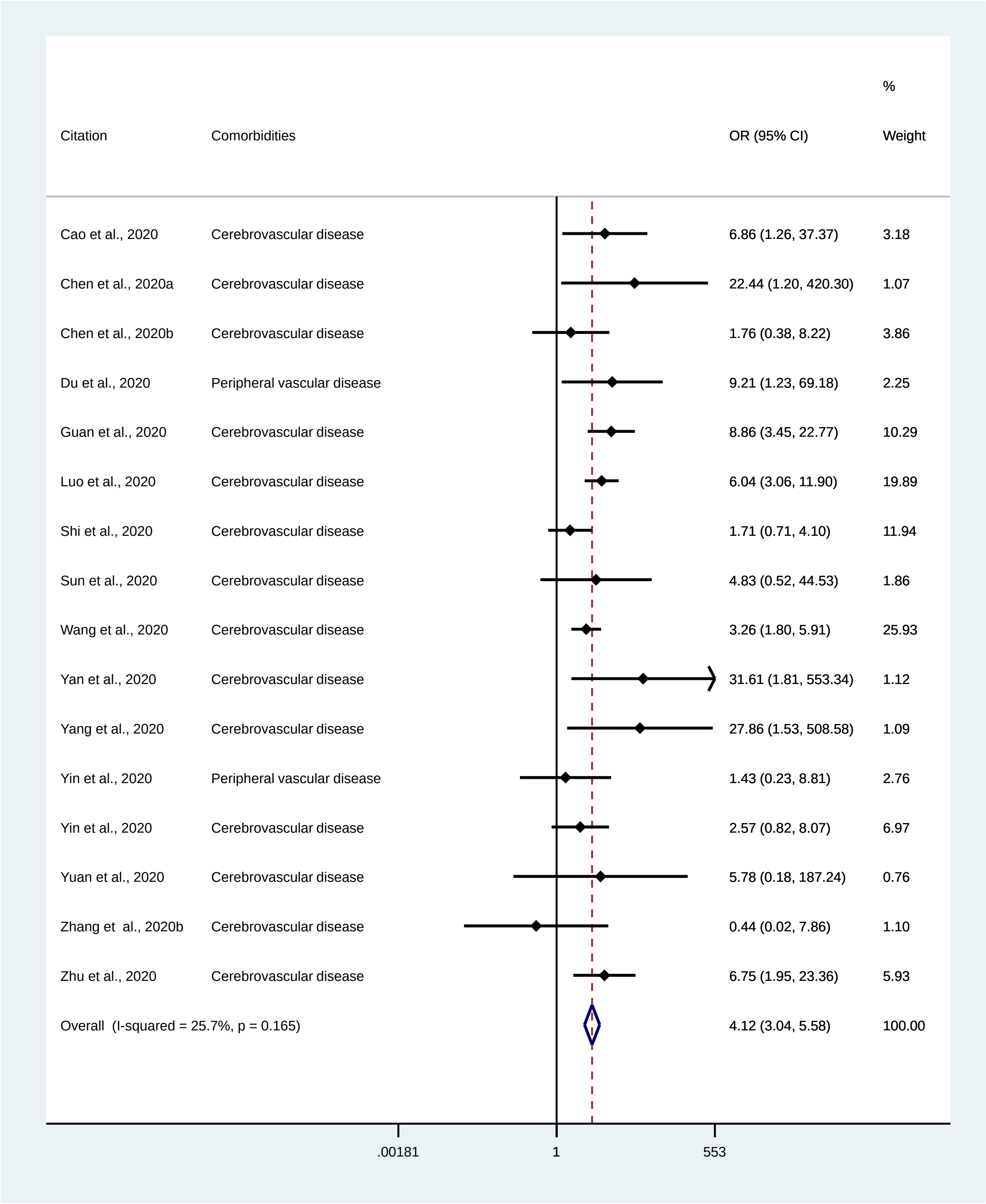

Figure S5. Likelihoods of death among patients with cerebrovascular system diseases patients infected further with COVID-19 disease

(a)

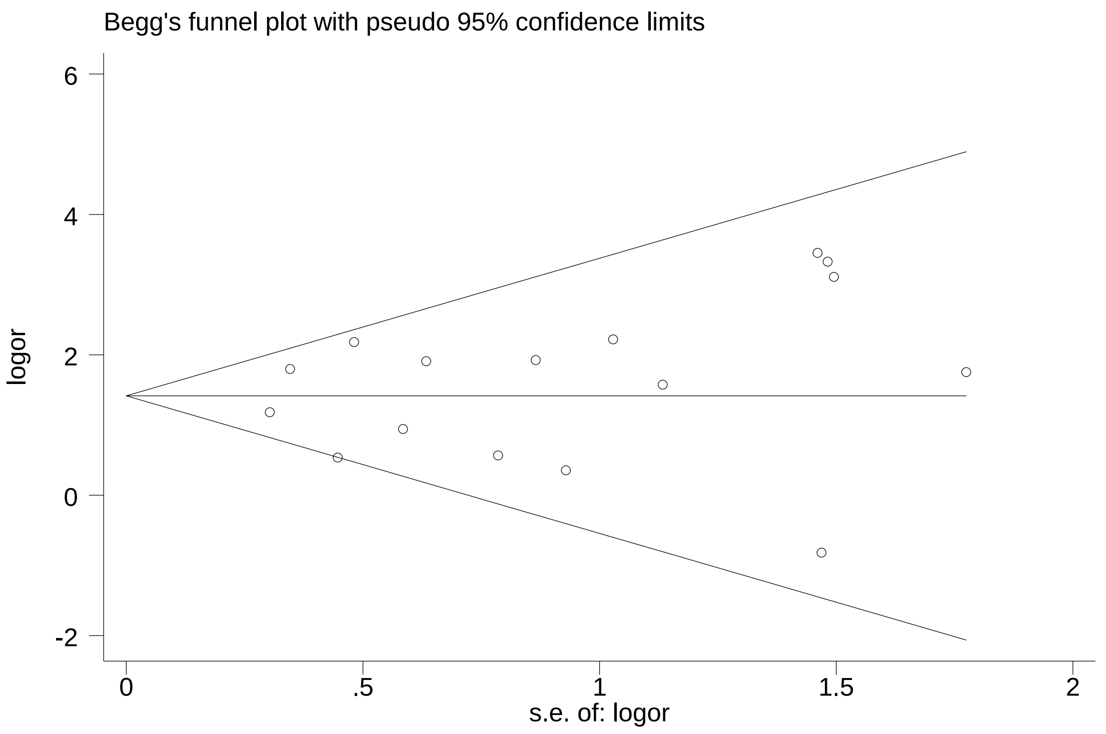

(b)

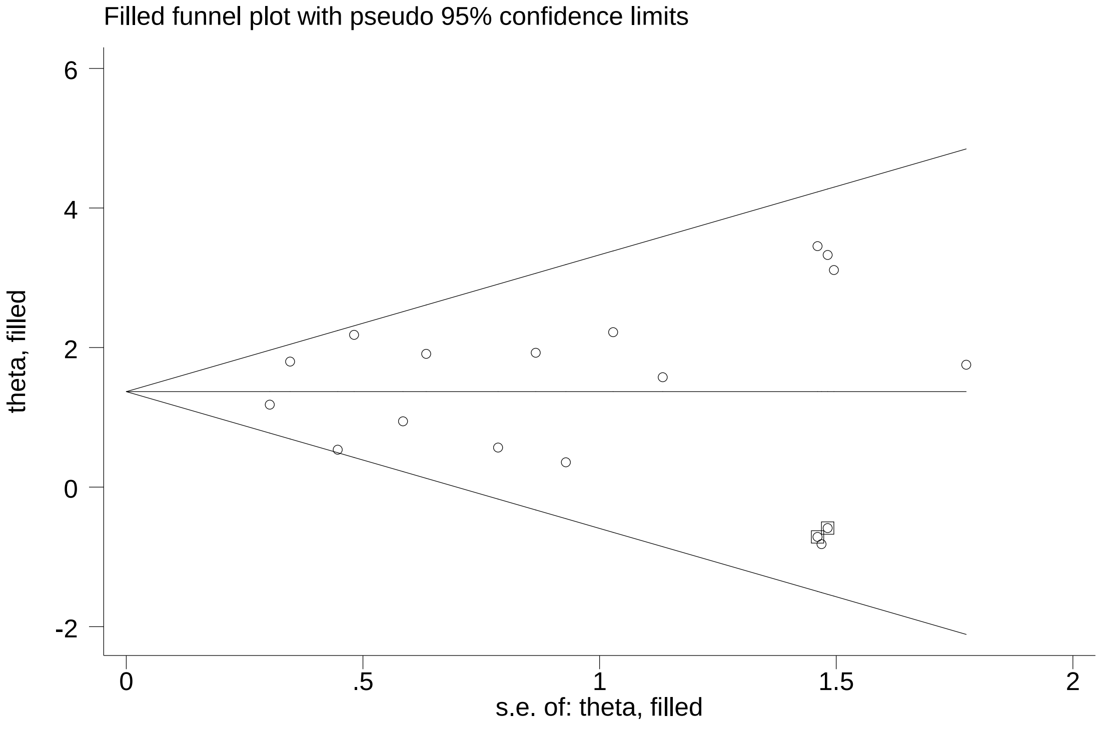

Figure 5a. Funnel plot without (a) and with trim and fill (b) estimate for Cerebrovascular system diseases patients infected further with COVID-19 disease

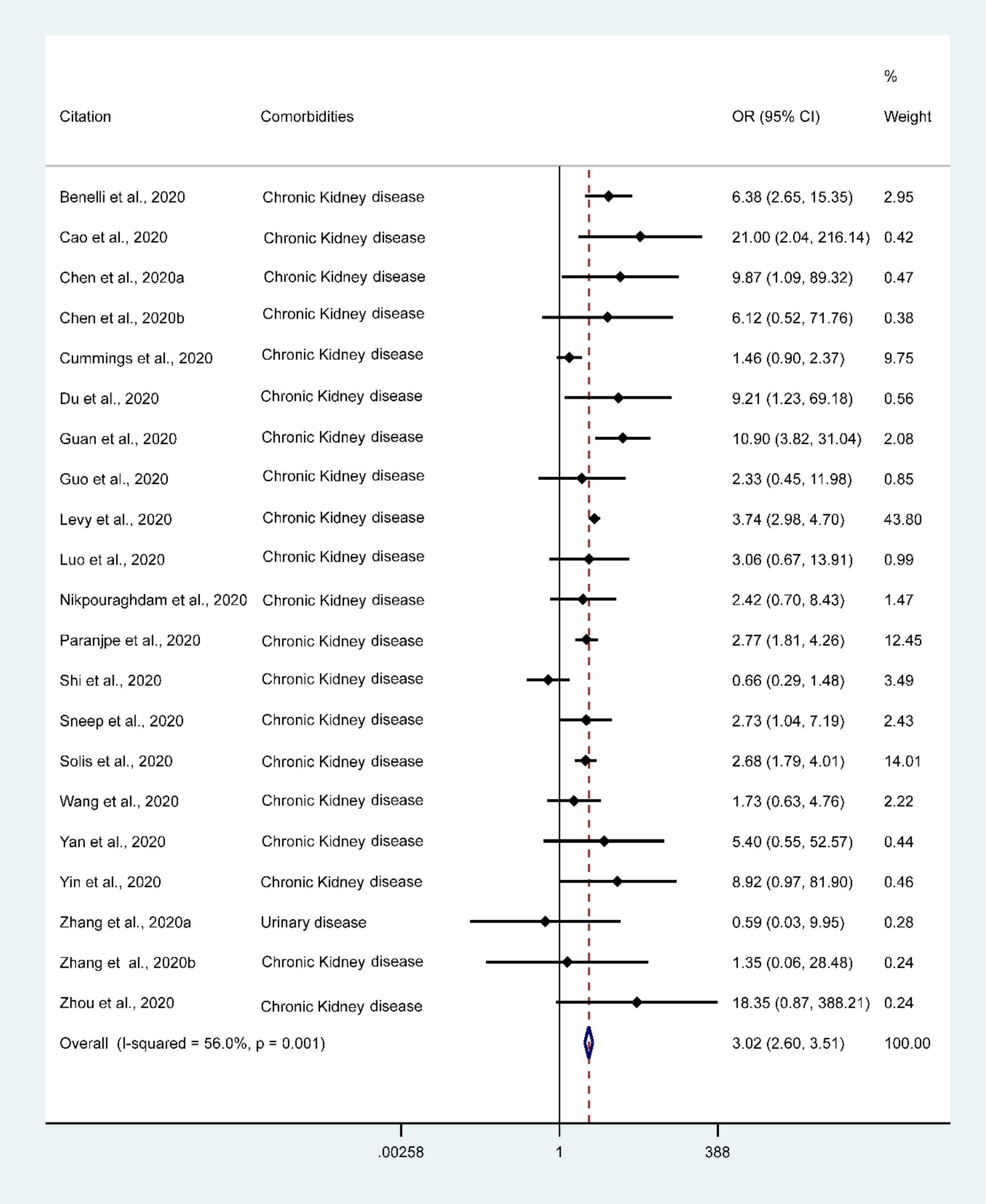

Figure S6. Likelihoods of death among patients with renal system diseases infected further with COVID-19 disease

(a)

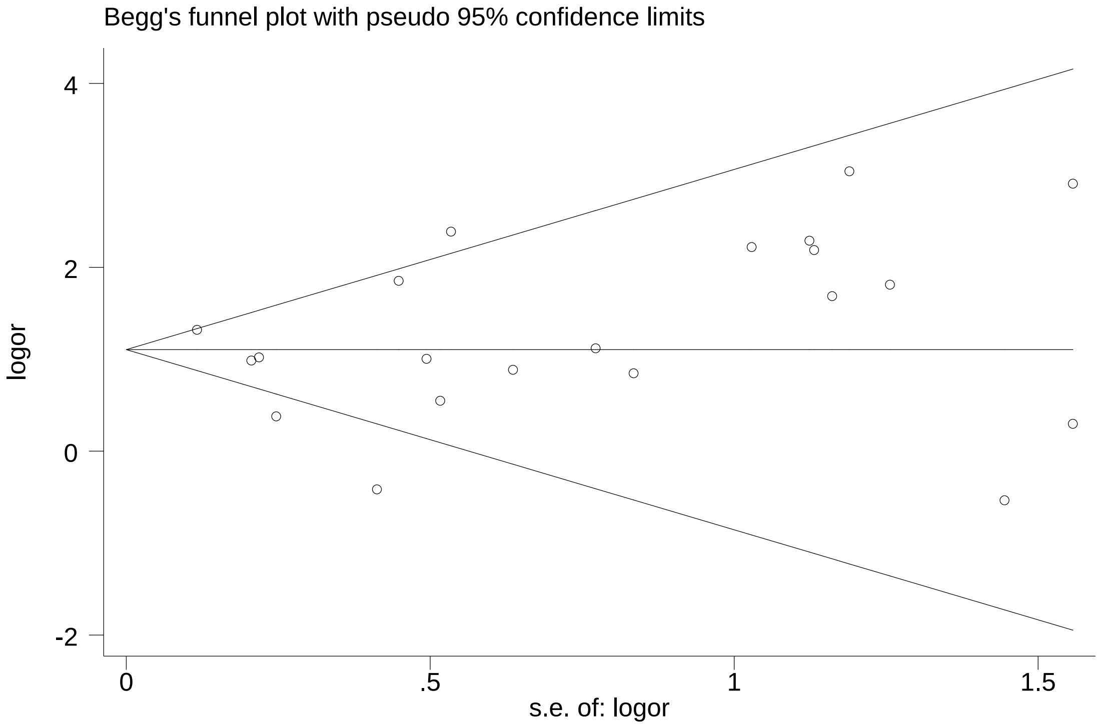

(b)

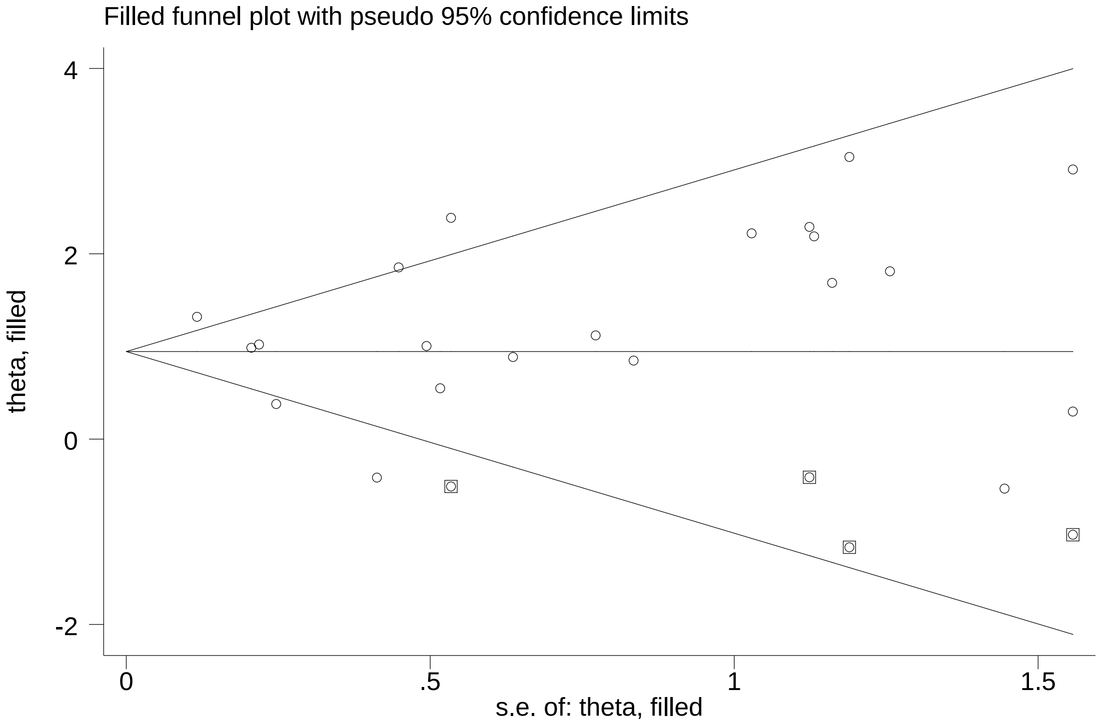

Figure S6a. Funnel plot without (a) and with trim and fill (b) estimate for renal system diseases patients infected further with COVID-19 disease

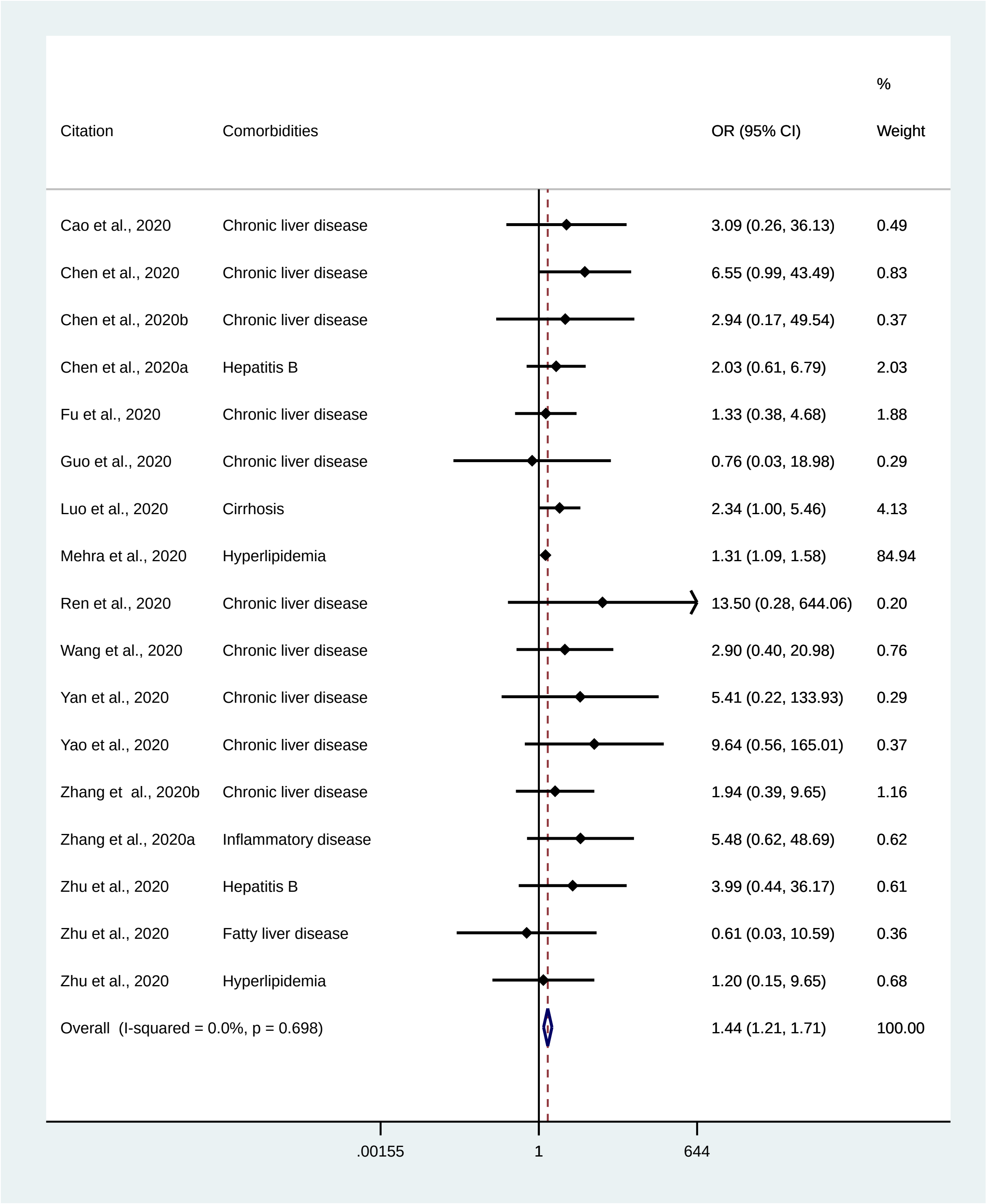

Figure S7. Likelihoods of death among patients with existing liver system diseases patients infected further with COVID-19 disease

(a)

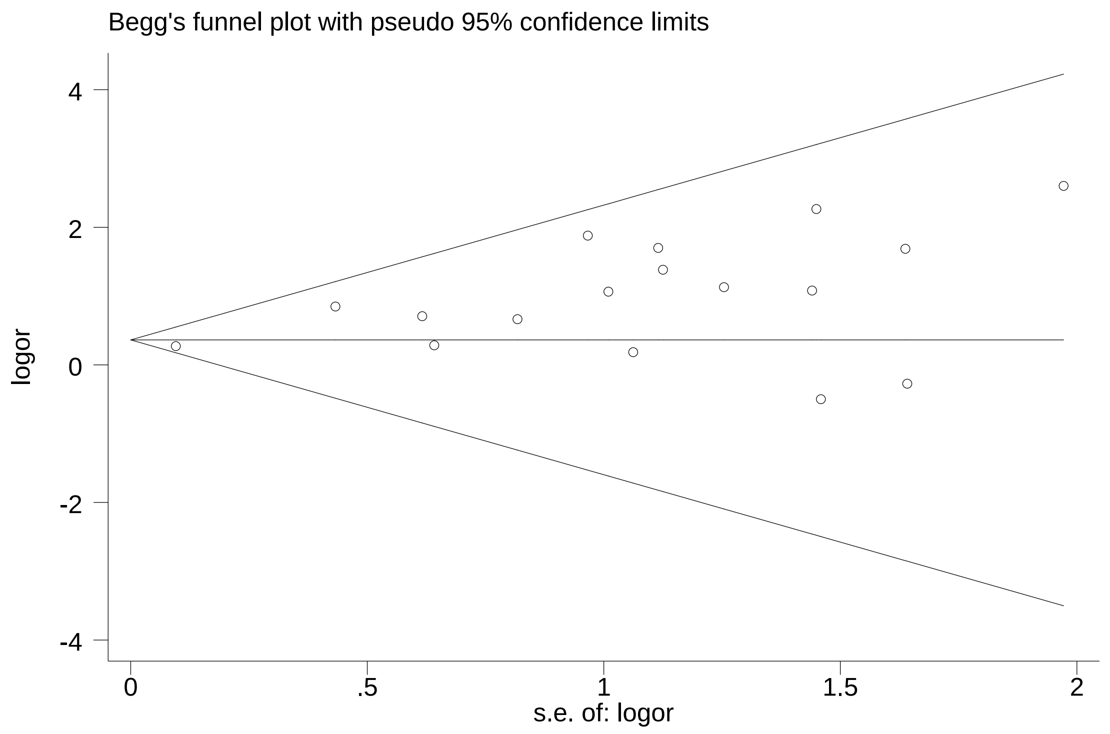

(b)

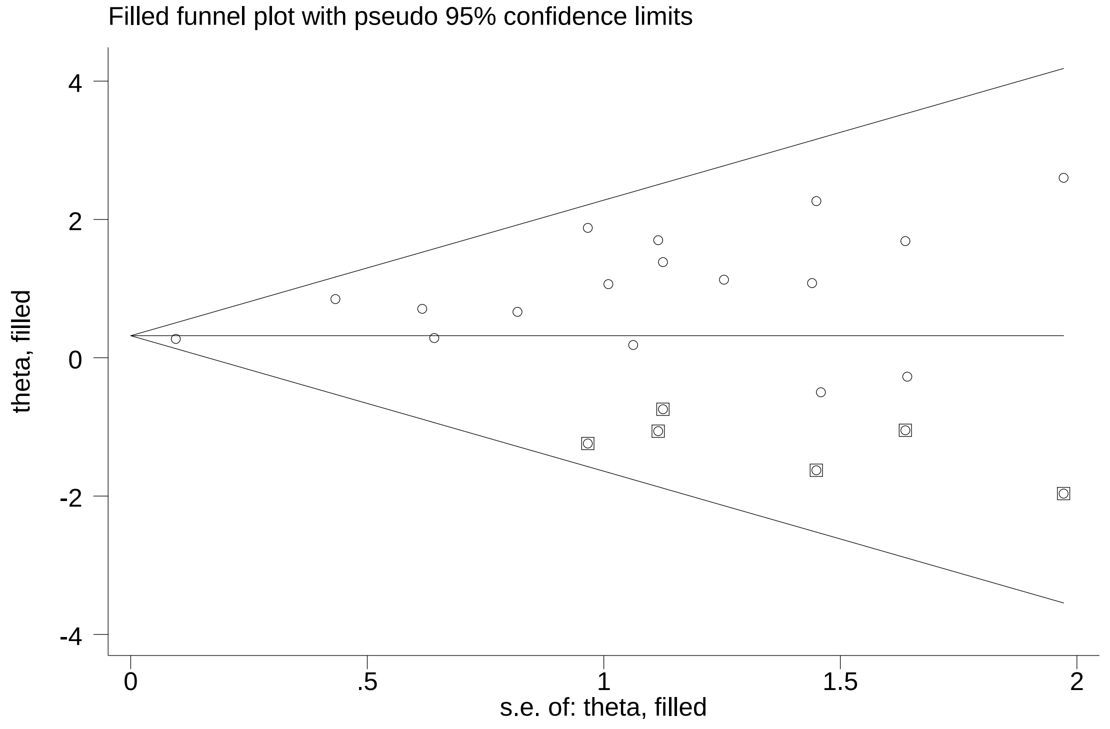

Figure S7a. Funnel plot without (a) and with trim and fill (b) estimate for liver system diseases patients infected further with COVID-19 disease

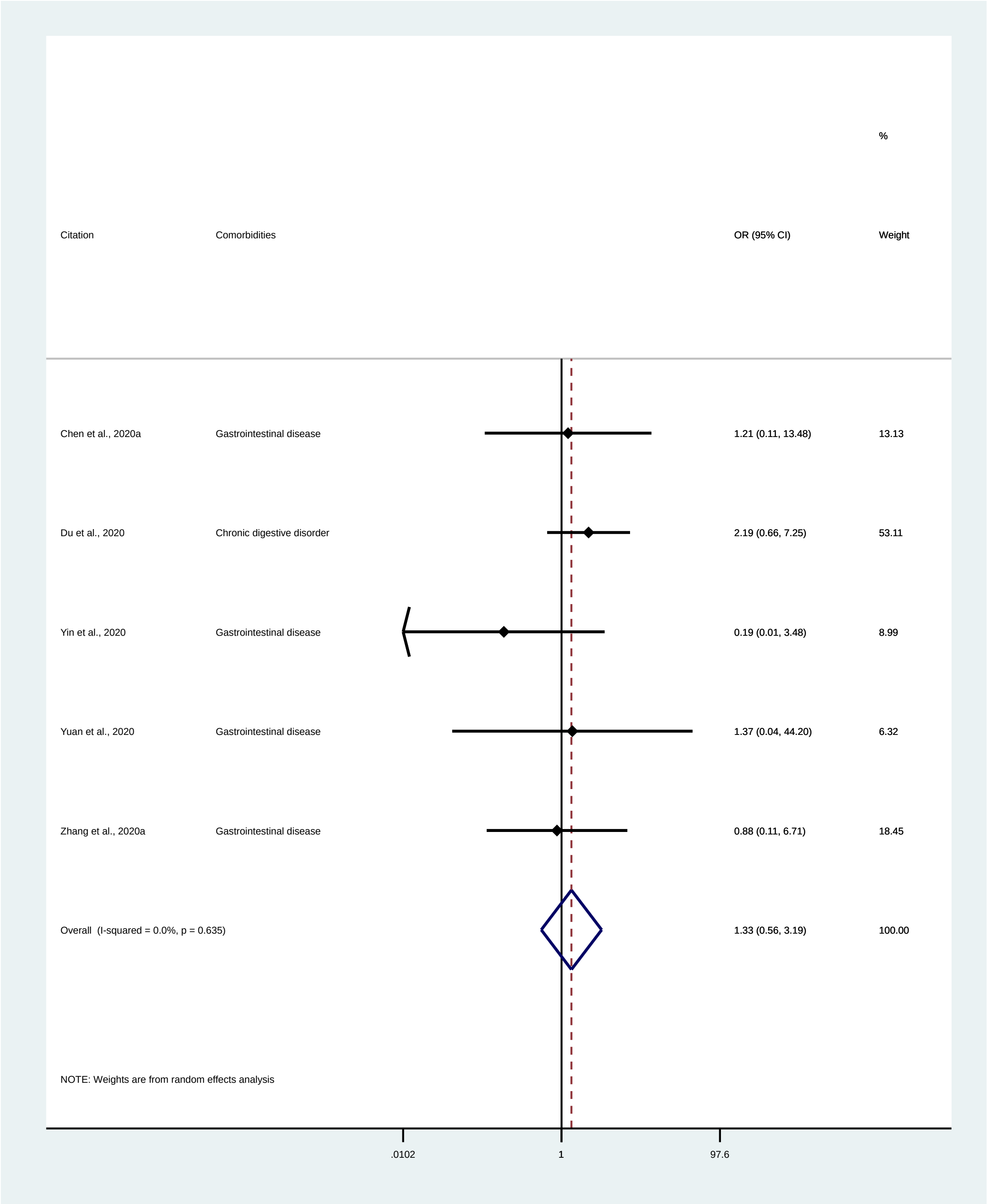

Figure S8. Likelihoods of death among patients with existing gastrointestinal system diseases patients infected further with COVID-19 disease

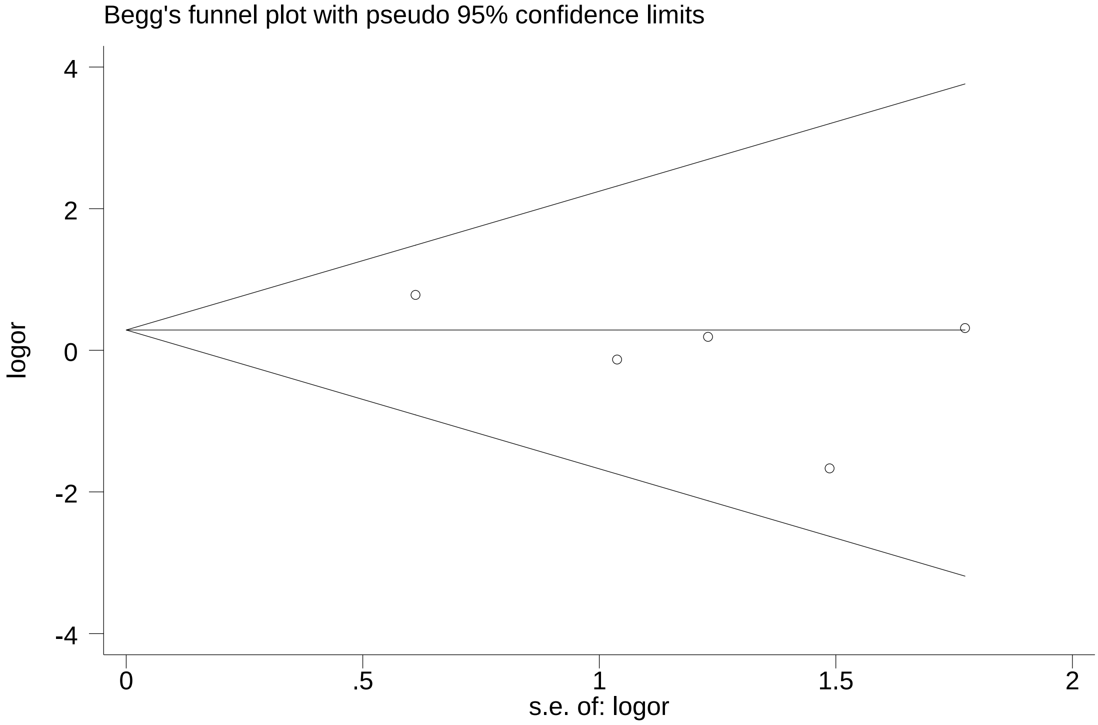

Figure S8a. Funnel plot for gastrointestinal system diseases patients infected further with COVID-19 disease
